# The Role of Polyphenols on Cognitive Function and Dementia Through Gut–Microbiota–Brain Axis Modulation: A Narrative Review

**DOI:** 10.3390/nu18111697

**Published:** 2026-05-26

**Authors:** Oualid Sbai, Aishwarya Chaure, Lorena Perrone, Patrick Poucheret

**Affiliations:** 1 Laboratory of Transmission, Control and Immunobiology of Infections (LTCII), LR11IPT02, Institut Pasteur de Tunis, Tunis 1068, Tunisia; oualid.sbai@pasteur.tn; 2 Department of Food and BioResource Technology, Faculty of Tropical AgriSciences, Czech University of Life Sciences Prague, 165 00 Prague, Czech Republic; chaure@ftz.czu.cz; 3 Department of Life Sciences, Health and Health Professions, Link Campus University, 00165 Rome, Italy; 4 Institute of Endotypes in Oncology, Metabolism and Immunology “G. Salvatore” (IEOMI), Consiglio Nazionale delle Ricerche (CNR), 80100 Naples, Italy; 5 Univ Montpellier, CIRAD, Montpellier SupAgro, Univ Avignon, Univ La Réunion, IRD, 34090 Montpellier, France; patrick.poucheret@umontpellier.fr

**Keywords:** polyphenols, diet, cognitive decline, neurodegeneration, gut microbiota, gut–microbiota–brain axis

## Abstract

The number of individuals affected by dementia and cognitive decline is progressively increasing, becoming a serious global health challenge. Several investigations underline the role of nutrition and dietary habits as a preventive strategy. Recent studies suggest that dietary supplementation with polyphenols may constitute an efficient preventive strategy. Indeed, it is emerging that polyphenols exhibit a neuroprotective effect because of their pronounced antioxidant and anti-inflammatory activity. Notably, several studies underline the role of the gut microbiota in the metabolism of the polyphenols, producing bioactive molecules that are absorbed through the gastrointestinal tract. They may exhibit beneficial effects on the central nervous system. Moreover, dietary polyphenols modulate gut microbiota composition, demonstrating a reciprocal regulation between gut microbiota and polyphenol-induced effects on brain functions. Thus, polyphenols are proposed to have an important role on the gut–microbiota–brain axis regulation. The literature search for this narrative review was conducted across three electronic databases PubMed, Scopus, and Web of Science as well as the NIH ClinicalTrials.gov registry, covering the period from January 2000 to 10 February 2026. The following search terms were used: “polyphenols”, “microbiota”, “gut–brain axis”, “dementia”, “cognitive function”, “polyphenols and cognitive dysfunction”, and “polyphenols and microbiota”. The study selection process was performed in two sequential stages: (i) screening of titles and abstracts, followed by (ii) full-text assessment for eligibility. Articles were included if they were peer-reviewed studies (in vitro, in vivo, or clinical trials), published in English, and addressed the effects of polyphenols on cognitive outcomes, gut microbiota composition, or the gut–microbiota–brain axis. Exclusion criteria included non-peer-reviewed sources, studies lacking relevant cognitive or microbiota-related endpoints, and publications not available in full.

## 1. Introduction

The characteristics of dementia syndrome are cognitive dysfunction, including memory loss, reduced executive functions, language impairment, and affected performance in daily life activities [1]. Dementia affects approximately 55 million individuals worldwide, and its prevalence is expected to rise up to 131.5 million by 2050, representing a major global health challenge [2,3]. Notably, about 70% of older people are living in low–middle income countries [3], representing a significant economic weight associated with medical costs for health systems [3]. Moreover, dementia represents a significant social burden because dementia produces social exclusion and affects not only the patients but also their relatives and caregivers [4]. Thus, dementia represents an increasing issue for the health system as well as for society. Indeed, dementia exerts a massive socio-economic impact not only because of the financial expenditure in terms of health medications and treatments, but also because it affects the emotional and physical well-being of patients’ family members and caregivers in particular, by lowering their productivity [5,6].

Although the prevalence of dementia is growing, frequently the diagnosis of cognitive decline is delayed, underestimated, and underdiagnosed [1]. The post-diagnostic therapy of people with dementia exhibits several difficulties, complex services and limited efficacy of the therapy [7]. The World’s Alzheimer report (2022) strongly encourages a personalized treatment and care of people affected by dementia, which must consider the personal needs of the patients [7]. In order to provide personalized care and effective therapy, it is necessary to improve the early diagnosis of cognitive decline. In addition, it is necessary to unveil the risk factors promoting dementia in order to develop preventive strategies. Indeed, the onset and progression of dementia and diseases characterized by cognitive impairment are modulated by lifestyle, genetic and environmental factors [8]. Thus, several studies explored the efficacy of innovative therapeutic strategies, not limited to the use of pharmaceutical compounds [9]. Interestingly, some of the lifestyle-associated factors are modifiable, such as diet and exercise, opening the way for a preventive strategy [10].

Recent investigations underline the relevance of the gut–microbiota–brain axis in the development of neurodegenerative diseases and dementia. The gut microbiota is composed of trillions of microorganisms cohabiting in the intestine, while the microbiome includes these microorganisms, their genes, the functions of these genes and their interaction with the environment. Recent evidence highlights the importance of the gut microbiota in modulating brain function and neurodegeneration through the gut–microbiota–brain axis [11]. Alterations of the gut microbiota, defined as dysbiosis, promote cognitive dysfunction by affecting metabolic equilibrium and inducing oxidative stress and inflammation [12]. It has been shown that gut dysbiosis promotes the onset and progression of Alzheimer’s disease (AD) as a consequence of altered gut microbiota metabolites that lead to the production of gut neurotoxins and the induction of the immune system [13]. In agreement, several studies demonstrated that dysbiosis plays an essential role in promoting and maintaining a systemic inflammatory status, which in turn affects brain functions, contributing to several neurodegenerative diseases, including those characterized by dementia [14]. Recent studies demonstrated that a bidirectional regulation occurs between the gut and the brain and that gut microbiota plays a key role in this bidirectional modulation [15,16]. These recent findings strongly suggest that a therapeutic approach targeting gut microbiota could be beneficial for the prevention of dementia, potentially slowing down its progression [17]. Indeed, several studies underlined the role of nutrition in modulating the gut–microbiota–brain axis in various brain diseases associated with dementia, such as Huntington’s disease (HD) [18], AD and Parkinson’s disease (PD) [17].

Defined dietary habits and nutrients have a beneficial effect on several neurological diseases, including dementia [19,20,21,22]. Dietary habits seem to exhibit a key function in gut–microbiota–brain axis modulation, which in turn has an impact on the onset and progression of cognitive dysfunction [23,24]. In particular, the Mediterranean diet (MeDi) and Mediterranean lifestyle showed a beneficial role in gut microbiota modulation of prophylaxis and progression of cognitive decline [9,25,26]. MeDi is characterized by the high consumption of specific types of foods, such as olive oil as a prevalent source of fat [27,28,29]. Indeed, extra virgin olive oil exhibits a beneficial effect against neurodegenerative diseases [30]. Notably, MeDi and extra virgin olive oil are enriched in polyphenols [31]. Several studies demonstrate that polyphenols are beneficial against cognitive decline [32,33]. Notably, polyphenols improve gut dysbiosis through promoting the proliferation of beneficial microbe strains and the production of specific microbiota metabolites that act positively on the gut–brain axis [34]. Indeed, polyphenols are suggested for the prophylaxis and therapy of cognitive impairment by modulating the gut–microbiota–brain axis [35].

## 2. Cognitive Function, Dementia and Dementia-Associated Diseases

### 2.1. Definition of Cognitive Function

Cognitive function encompasses a broad range of brain processes, including memory, executive function, attention, language, and learning capacity, all of which contribute to knowledge acquisition, information processing, and interpretation [36]. Impairments in these domains are considered early indicators of neurodegenerative disorders such as Alzheimer’s disease and Parkinson’s disease. Cognitive decline is a defining feature of neurodegenerative diseases and dementia, involving the progressive deterioration of memory, executive function, and other higher-order brain processes. Moreover, cognitive decline is now understood to be influenced not only by aging and genetic predisposition but also by environmental and dietary factors, notably gut microbiota composition and polyphenol intake. The Diagnostic and Statistical Manual of Mental Disorders, Fifth Edition (DSM-5) provides a standardized diagnostic framework for neurodegenerative disorders, distinguishing mild cognitive disorder (formerly mild cognitive impairment, MCI) from major neurodegenerative disorder (formerly dementia) based on the severity of decline and its impact on daily independence [37]. The DSM-5 provides a classification of the six domains involved in cognitive function [38], and composed of complex attention, executive function, learning and memory, language, perceptual-motor control, and social cognition [38]. Critically, the DSM-5 requires that cognitive deficits should not be better explained by a psychiatric disorder. Conditions such as major depressive disorder can produce reversible hippocampal volume loss and pseudodementia that mimic early neurodegenerative dementia, underscoring the clinical importance of differential diagnosis [39]. The pathological basis of dementia extends beyond neuronal loss to encompass the dysfunction of glial cells, astrocytes and microglia whose altered interactions with neurons drive neuroinflammation, synaptic failure, and progressive cognitive decline in Alzheimer’s disease, Parkinson’s disease, and related disorders.

The brain is organized into three main structures: the cerebrum, cerebellum, and brainstem, with the cerebrum playing a central role in cognition. The cerebral cortex is divided into four lobes with specialized functions. The frontal lobe, particularly the prefrontal cortex, is involved in executive functions, decision-making, working memory, and behavioral regulation [40,41]. The parietal lobe contributes to sensory integration and spatial processing, while the temporal lobe, including the hippocampus, is essential for memory and language comprehension. The occipital lobe is primarily responsible for visual processing. In addition, subcortical structures such as the cerebellum, thalamus, and basal ganglia contribute to motor coordination, sensory relay, and procedural learning. Overall, cognitive functions rely on coordinated activity across frontal, parietal, and temporal regions, particularly the prefrontal cortex and medial temporal areas, which are also affected during age-related cognitive decline [42].

### 2.2. Dementia and Associated Disorders

Cognitive disorders are classified on the basis of the etiology. The classification in subtypes is the same for both mild and major cognitive disorders. Indeed, after the neuropsychiatric assessment, further analysis is required in order to unveil the specific etiology of the disease [38]. For several diseases there are well characterized tests that can identify the etiology, such as Parkinson’s disease (PD), Huntington’s disease (HD), HIV infection and AIDS, traumatic brain injury, and stroke. Regarding other disorders, the clinical assessment precedes the definition of the etiology, such as Alzheimer’s disease (AD), frontotemporal dementia, dementia with Lewy bodies, and vascular dementia [37]. However, the discovery of new biomarkers for these diseases is improving, allowing an early assessment of the etiology.

Alzheimer’s disease (AD) is a neurodegenerative disease with a gradual and progressive cognitive decline, particularly in the learning and memory functions. It is characterized by the presence of amyloid plaques and neurofibrillary tangles [43]. According to the DMS-5, the diagnosis of major cognitive decline in AD requires a deficit in two cognitive domains, with impairment of learning and memory as one of the two. For the diagnosis of mild cognitive impairment, the deficit in learning and memory is sufficient [38]. AD may be genetically inherited and caused by mutations of genes producing high levels of amyloid beta peptide (Aβ): the amyloid precursor protein (APP), the presenilins, PSEN1 and PSEN2 [43]. However, more than 95% of AD cases are sporadic [44]. It seems that the oligomeric forms of β are the most toxic [45,46]. Notably, several risk factors have been described for AD, such as the presence of defined APOE alleles [47]. The diagnosis of AD is improving by the characterization of biomarkers, such as APOE alleles, the level of Aβ and tau in the cerebrospinal fluid, the detection of amyloid in the brain by imaging, such as positron emission tomography (PET) and new biomarkers currently under validation [37]. Recently, a neuroinflammatory hypothesis for AD etiology was suggested [48,49,50]. This theory is further supported by several data demonstrating the role of dietary patterns and nutrition in promoting AD prevalence by promoting metabolic alterations and inflammation [21,51,52]. These data strongly suggest that a dietary intervention may be beneficial for the prevention of AD and may be used as therapeutic support for the cure of AD.

Vascular dementia (or vascular neurocognitive disorder) describes a spectrum of cognitive diseases having as predominant etiology cardiovascular alterations, including ischemic and hemorrhagic injury, as well as alterations induced by small vessel diseases. The diagnosis of vascular neurocognitive disorder can occur even in the absence of a history or indications of a previous stroke, and in this case, the etiology is considered to be due to small-vessel disease [38]. The cognitive alterations are prevalent alterations in processing speed and frontal-executive functions. The clinical symptoms also include hemiparesis, visual field defects, and pseudobulbar palsy, which are due to stroke or small vessel disease. Diagnosis is confirmed by neuroimaging analysis using magnetic resonance imaging (MRI), revealing the presence of multiple lacunar infarcts or large convergent white matter lesions [38]. There are no validated biomarkers for these disorders; thus, the diagnosis is confirmed only by neuroimaging analysis. However, recent studies unveiled several risk factors for vascular dementia [53]. Moreover, defined dietary patterns and nutrients can lower the risk of vascular dementia [54]. In particular, dietary flavonoids reduce the risk of vascular dementia [55], suggesting that nutrients can be beneficial for the prevention of this disease.

Parkinson’s Disease (PD) is a neurodegenerative disease characterized by motor symptoms, such as bradykinesia and resting tremors. PD also exhibits non-motor symptoms, comprising cognitive dysfunction, sleep disorders, psychological manifestations, enteric nervous system alterations, and constipation [56]. A distinctive hallmark of PD is the degeneration of dopaminergic neurons in the *basal ganglia* and *substantia nigra pars compacta* [57]. At the histological level, PD is characterized by the presence of α-synuclein aggregates, named Lewy bodies [58]. There are familial forms of PD, due to mutations in various genes that lead to the Mendelian inherited forms of PD: (i) typical recessive mutations in PARK2, PARK 7 and PINK1; or (ii) atypical recessive mutations in ATP13A2, PLA2G6, FBXO7: autosomal dominant mutations in the genes SNCA, LRRK2, VPS35, GBA [59]. These mutations affect various cellular mechanisms, promoting mitochondrial dysfunction [60], oxidative stress [61], neuroinflammation [62], and alterations in the ubiquitin/proteasome system (UPS) [63], ultimately inducing α-synuclein misfolding and aggregation, leading to synaptic dysfunction and neuronal apoptosis [64]. Familial forms of PD are quite rare. However, other genetic alterations have been discovered, which participate in PD onset and progression without a Mendelian inheritance [65]. The majority of PD cases are sporadic and it has been demonstrated that environmental factors, such as chemicals, increase PD risk [66]. Cognitive dysfunction correlates with a bad diagnosis at every PD stage. The early diagnosis of cognitive function in PD is possible by following the patient’s history, the executive function impairment, the visuospatial cognitive assessment, and testing memory capability. Moreover, various cerebrospinal fluid (CSF) biomarkers are available for the diagnosis of dementia and its progression in PD, as well as electroencephalogram (EEG) analysis [67,68]. Various symptoms that can underline the presence of cognitive dysfunction in PD, the most frequent are affected executive functions, memory impairment, and psychological alterations such as anxiety and depression, sleep alterations, and apathy [38]. It has been revealed that about 40 percent of PD patients are affected by MCI that parallels the motor and psychological symptoms, which can be considered as prognostic elements of dementia in PD [69,70]. Recently, various CSF and blood-derived biomarkers, as well as lipidomic data, have been demonstrated to be useful for the diagnosis and prognosis of cognitive impairment in PD [71]. Notably, PD is characterized by various non-motor symptoms, such as chemosensory impairment. Indeed, olfactory and taste alterations occur early in PD, leading to malnutrition and reduced appetite, which in turn lead to depression, further affecting the quality of life in PD subjects [72]. Recent studies also link bitter taste receptors to the misfolding of α-synuclein, and various researchers have demonstrated the role of gut microbiota composition in PD progression [72]. Thus, increasing evidence underlines the key role of diet in PD and the relevance of a healthy diet as a therapeutic strategy in PD patients [72]. Moreover, some evidence suggests that a nutritional treatment with polyphenols may be beneficial for the prevention and adjuvant therapy of PD [73].

### 2.3. Neuron–Glia Interactions in Neurodegeneration and Dementia

The central nervous system (CNS) comprises neurons and glial cells, primarily astrocytes and microglia, whose coordinated interactions are essential for maintaining neuronal homeostasis and cognitive function. In a healthy brain, astrocytes provide metabolic and trophic support to neurons, regulate glutamate clearance and ion homeostasis, maintain blood–brain barrier (BBB) integrity, and modulate synaptic transmission through gliotransmitter release. Microglia, the resident innate immune cells of the CNS, continuously survey brain parenchyma, phagocytose cellular debris and misfolded proteins, regulate synaptic pruning, and support neuronal survival through anti-inflammatory and neurotrophic signaling. Together, these neuron–glia interactions constitute a dynamic neuroprotective network that is critically disrupted in neurodegenerative diseases.

In AD, PD, LBD, and related dementias, microglial cells transition from a homeostatic surveillance state to a pro-inflammatory (M1) phenotype, releasing cytotoxic mediators including interleukin-1β (IL-1β), tumor necrosis factor-α (TNF-α), IL-6, and reactive oxygen species (ROS) that amplify neuronal damage and drive progressive cognitive decline [50]. This microglial-driven neuroinflammation is now recognized as a primary pathological driver rather than a secondary consequence of neurodegeneration. Concomitantly, astrocytes undergo reactive astrogliosis and adopt a neurotoxic A1 phenotype, impairing synapse formation, reducing neurotrophic factor secretion (notably BDNF and GDNF), and amplifying neuroinflammatory cascades. In AD, microglia surrounding amyloid-β (Aβ) plaques initially attempt phagocytic clearance, but sustained activation leads to clearance failure and entrenched neuroinflammation. Reactive astrocytes associated with Aβ deposits further impair synaptic function, contributing to the synaptic loss that correlates most strongly with cognitive decline [48]. In PD and DLB, microglial and astrocytic activation in response to α-synuclein aggregates generates a self-amplifying neuroinflammatory loop that accelerates dopaminergic and cortical neurodegeneration. Importantly, polyphenols including resveratrol, curcumin, and quercetin have been shown to suppress microglial NF-κB activation and NLRP3 inflammasome assembly, promote microglial transition toward anti-inflammatory (M2) phenotypes, reduce reactive astrogliosis, and restore astrocytic neuroprotective functions. Microbiota-derived metabolites of polyphenols, including urolithins, ferulic acid, and short-chain fatty acids (SCFAs), cross the BBB and directly modulate glial cell activity, reinforcing the gut–microbiota–brain axis as a key mediator of polyphenol-driven neuroprotection in dementia.

## 3. Gut Microbiota and Gut–Brain Axis

### 3.1. Definition of Microbiota–Gut–Brain Axis

**Figure 1:**
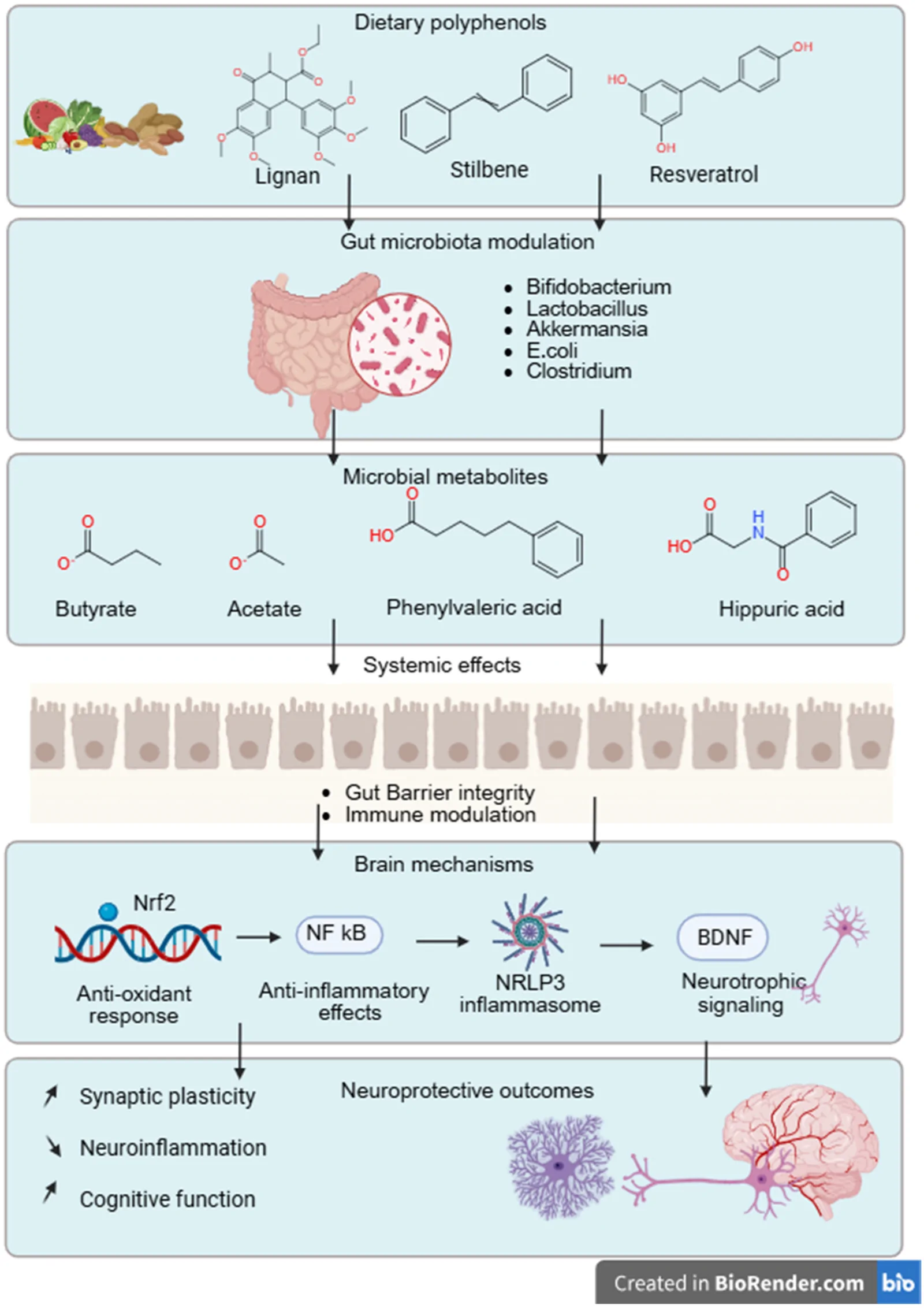
Schematic representation of the polyphenol–microbiota communication and its effect on the brain function.

The gut–brain axis connects emotional and cognitive functions with the function of the intestine. Indeed, evidence reveals a bidirectional complex communication network that links the gastrointestinal tract and the nervous system, including the CNS, the autonomic nervous system (ANS), the enteric nervous system (ENS) and the hypothalamic–pituitary–adrenal (HPA) axis [74]. The gut microbiota plays an essential role in the modulation of the gut–brain axis. The microbiota–gut–brain axis is an active interaction connecting various tissues and explains the mutual reciprocal influence of gut and brain that plays an essential role in human physiology and pathophysiology. Gut microbiota, a complex community of microorganisms, populates the gastrointestinal tract and exhibits a close symbiotic relationship with the host, playing a crucial role in health maintenance, allowing the metabolism of indigestible dietary components and the synthesis of some vitamins, preventing pathogen colonization, and contributing to the maturation and education of the immune system [75]. The microbiota is distributed in the human gastrointestinal tract and although each subject’s microbiota profile is different, the relative abundance and distribution along the intestine of these bacterial phylotypes are similar among healthy individuals. Firmicutes and Bacteroides are the major phyla of the microbiome, about ¾ of the microbiome [76]. This community exerts an important metabolic and physiological role for the host and contributes to its homeostasis during life (Figure 1). 

### 3.2. Composition and Roles of the Gut Microbiota: Effect on the Gut–Brain Axis

Although the gut and brain are separated and distant in the body and belong to two different systems, preclinical and clinical studies have shown several pathways that connect the gut microbiota with CNS function [74]. The structure and function of the brain can be regulated by the gut; on the other hand, the brain controls the microbiota microenvironment and composition. The complexity of these interactions plays a role in gut functions, as well as linking emotional and cognitive centers of the brain with peripheral intestinal functions, such as immune activation, intestinal permeability, enteric reflex, and entero-endocrine signaling [74]. Growing evidence demonstrates that the gut–brain axis is strongly modulated by the microbiota. The role of the microbiota is not limited to the protection of gastrointestinal homeostasis but also exhibits multiple effects on other body functions like affect, motivation, and cognition. This implies that the gut microbiota could be a promising therapeutic target for the modulation of diverse physiological and psychological processes like mood, cognition, and stress response [77]. The gut microbiota is a complicated ecosystem containing a massive quantity of microorganisms. The human gastrointestinal tract is colonized by the most complex and diversified microbial community in the human body, including bacteria, fungi, viruses, and archaea [78]. More than 2000 bacterial species have been identified in the human gut and it has been estimated that the gut microbiota contains nearly 150 times more genes compared to the human genome. Bacteria are the most functionally important group and play an important role in digestion, immune function, and neurological function [79,80,81]. The human gut microbiota contains four major bacterial phyla, including Firmicutes, Bacteroidetes, Actinobacteria, and Proteobacteria [82]. The diversity of gut microbiota, determined by the relative abundance of different bacterial phyla, is essential for maintaining gut homeostasis and overall health [83]. Bacteria participate in digestion by breaking down complex carbohydrates and fibers that the human digestive tract cannot assimilate and producing short-chain fatty acids (SCFAs) like butyrate, which are an energy source for colon cells. The gut microbiota also synthesizes vitamins, like vitamin K and some B vitamins, which are then absorbed into the bloodstream. Notably, gut bacteria play an important role in the early stages of life, supporting the development of the immune system in newborns. In adult persons, gut microbiota regulates immunity, auto-immunity and intestinal barrier integrity preservation [84]. Bacteria are also important for protecting the human body from pathogens. Indeed, bacteria prevent the colonization of pathogens and reduce the availability of nutrients for pathogens. Moreover, bacteria inhibit pathogens’ growth and can also induce their death. Gut microbiota modulates the immune responses by controlling the development and function of various immune cells, including T cells, B cells, and macrophages [85]. The gut microbiota can support both pro-inflammatory and anti-inflammatory responses to maintain the immune system homeostasis. Recent studies demonstrate that dysbiosis can disturb the immune system homeostasis and contribute to various diseases like inflammatory bowel disease, autoimmune diseases, and cancers [86]. Recent studies underline the role of gut microbiota as a major determinant of plasma metabolome [81]. Several studies have shown that gut microbiota exhibits a significant impact on mental health and neuropsychiatric disorders [87]. Microbiota can exert opposite functions: *Bifidobacterium bifidum* and *Lactobacillus acidophilus* promote intestinal motility, while *Escherichia* species can inhibit it. Gut microbes communicate to the central nervous system through at least three parallel and interconnected pathways involving nervous, endocrine, and immune signaling mechanisms [88,89]. These pathways provide the central physiological connection between the brain and the gut. The gut communicates through releasing active substances, quorum sensing, and secondary metabolites [90]. The efferent brain–gut signaling contains neuroendocrine and neuroimmune mediators, as well as molecules produced by the autonomic systems [91]. The afferent gut–brain axis includes the enteroendocrine system, cytokines, sensory epithelial cells, and microbiota. Several studies using animal models like germ-free mice demonstrate the involvement of the microbiota in promoting PD in mice carrying mutant Glucosidase Beta Acid 1 (GBA). The microbiota modulates the stress reactivity and anxiety-like behavior and regulates the hypothalamic–pituitary–adrenal (HPA activity) in germ-free (GF) animals. Indeed, this animal model is characterized by reduced anxiety and an enhanced stress response with increased adrenocorticotropic hormone (ACTH) and cortisol levels [74,92,93,94,95,96]. The microbiota plays an important role in the development of the brain. Indeed, GF animals exhibit an altered expression and turnover of neurotransmitters into the brain, affecting the nervous system’s normal modulation [92,93,94,96]. Moreover, GF animals exhibit an alteration of the gut sensory-motor functions, with delayed gastric emptying and intestinal transit [97]. Preclinical studies demonstrate that dysbiosis due to antibiotic exposure or chronic bacterial infection affects brain function during early life or adulthood [98,99]. The gut microbiota can modulate several events in the brain, such as neurogenesis, myelination, dendritic spine morphology, microglia morphology, blood–brain barrier (BBB) structure and permeability, synapse structure and function. Adult neurogenesis is considerably regulated by the microbiome [100,101]. Neurogenesis in the dentate gyrus of the hippocampus is significantly reduced in germ-free (GF) mice and in C57BL/6 mice affected by dysbiosis due to vancomycin treatment [101,102]. Introduction of the microbiota derived from wild-type mice into the gut of GF mice promotes neurogenesis in these mice, compared to untreated GF mice. Interestingly, when the microbiota is introduced in GF mice lacking the Tryptophan hydroxylase 1 (TPH1), neurogenesis is strongly reduced, the serotonin synthesis is impaired, and the levels of neural precursors expressing Nesting are diminished [103].

Butyrate, a gut bacteria metabolite, has been shown to promote hippocampal neurogenesis in pigs. It is, therefore, hypothesized that butyrate-producing bacteria, such as *Faecalibacterium prausnitzii*, *Anaerobutyricum hallii*, *[Eubacterium] rectale*, *Lactobacillus casei*, *Coprococcus eutactus*, *Coprococcus comes*, *[butyricicoccus] pullicaecorum*, and *Clostridium butyricum* can modulate neurogenesis via the gut–brain axis [88,104,105,106].

Several studies have demonstrated a correlation between intestinal microbiota composition and myelin formation. Germ-free (GF) mice exhibit altered myelination patterns and reduced expression of myelin-related genes, while microbiota colonization restores myelin gene expression and white matter integrity [107,108]. In addition, microbiota-derived signals have been shown to regulate oligodendrocyte differentiation and myelin plasticity, further supporting the role of the gut–microbiota–brain axis in myelination processes [109].

Emerging evidences unveil that dendritic spine development is regulated by extrinsic factors [110]. These extrinsic factors include neurotransmitters and neurotrophins. However, recent data show that the microbiota has an important effect on dendritic structure. It seems that the microbiota can either protect or destroy dendritic development. GF mice exhibit aberrant mushroom-shaped dendrites, which appear thinner, shorter, and shrunken compared to control mice, leading to reduced synaptic strength and plasticity, despite enhanced dendritic branching. Indeed, administration of galactooligosaccharides (GOS), as a prebiotic agent, amplifies dendritic spine density in rats [111].

Several studies support the role of gut microbiota in modulating the integrity of the BBB [112,113,114]. Experimental studies unveil that mice with normal microbiota develop a BBB around postnatal day 14, while GF mice exhibit amplified BBB permeability and reduced endothelial tight junction proteins at postnatal day 16. Colonization of GF mice with pathogen-free microbiota restores the BBB integrity [112]. Gut colonization with *Lactobacillus plantarum*, a lactic acid-producing Gram-positive bacterium, increases BBB integrity in Swiss albino mice [113]. Moreover, administration of *Clostridium butyricum* following mice traumatic brain injury ameliorates neuronal degeneration and BBB permeability [114]. Emerging evidence indicates that alteration of the gastrointestinal (GI) barrier affects BBB permeability by promoting inflammation. These data are supported by the demonstration that bacterial metabolites such as butyrate and propionate enhance the integrity of the GI barrier by facilitating the assembly of tight junctions [115].

Several data suggest that gut microbes modulate synaptic plasticity. Indeed, hippocampal synaptic plasticity is modulated by glucocorticoids [116], which are metabolized by gut bacteria, such as *Eggerthella lenta*, *[Clostridium] scindens 1*, and *[Clostridium] scindens 2* [117]. These bacteria modulate synaptic function following neuronal injury [118], while it does not occur in GF mice.

Furthermore, *Lactobacillus casei* can reorganize the dentate gyrus (DG) neurogenesis, CA3-CA1 synaptic activity, and hippocampal brain-derived neurotrophic factor-Tropomyosin receptor kinase B (BDNF–TrkB) signaling in mice subjected to gut microbiota damage by a two-week antibiotic cocktail administration [119] and increase serotonin mRNA levels in juvenile rats’ DG [120]. The combination of a probiotic with a prebiotic (symbiotic), including *Lactobacillus casei* with inulin, enhances the activity of the serotonin receptor 5-HT1A in the hippocampus, particularly in the CA1and DG of healthy juvenile rats [120].

Accumulating evidence suggests that microbes within the gut are involved in brain morphology alterations. First, data obtained using GF animals demonstrate that brain morphology is impacted when the microbiota is absent [92,94]. Secondly, animals receiving defined and different strains of bacteria exhibit changes in different brain regions [121,122,123].

Microbiota participates in anxiety modulation through the HPA system by influencing brain neurochemistry. The HPA axis, a part of the limbic system, modulates memory and emotion to regulate the response to stress. The gut microbiota acts on the HPA axis through several, both direct and indirect, signaling pathways by: (i) producing LPS, neurotransmitters and metabolites, (ii) modulating cortisol levels and (iii) controlling gut permeability. This interaction influences various physiological processes and behaviors. The communication between microbiota and the brain engages the vagus nerve (VN), which transmits information from the luminal environment to the CNS [74].

### 3.3. Role of the Microbiota–Gut–Brain Axis on Cognitive Impairment

Alterations in the gut–microbiota–brain axis have been increasingly associated with cognitive impairment and neurodegenerative diseases. Clinical studies revealed that the diversity of the microbial species composing the gut microbiota is reduced in AD patients, suggesting that the microbiota may participate in the deterioration of cognitive functions [124]. In agreement, Zhaung and colleagues demonstrated that the microbiota derived from fecal samples of AD patients showed an altered composition [125]. A preclinical study analyzed the role of gut microbiota alterations on amyloid beta (Aβ) formation in the APPSWE/PS1ΔE9 mouse model of AD. The gut microbiota composition was altered by antibiotic treatment, unveiling that the modification of microbiota composition regulated host innate immunity, which in turn decreased Aβ production [126]. This study clearly demonstrated that a prolonged shift in gut microbial composition and diversity induced by long-term and broad-spectrum combinatorial antibiotic treatment regimes reduced Aβ plaque accumulation, while levels of soluble Aβ increased [126]. Gut microbiota composition affects the gut–brain axis, which modulates neuroinflammation and the progression of AD. Although the gut microbiota changes several times during the normal aging process, its composition in patients with neurodegenerative diseases displays a reduced microbial diversity and a shift in the abundance of specific bacterial taxa [127]. Five different gut bacterial taxa (*Erysipelatoclostridiaceae*, *Erysipelotrichales*, *Patescibacteria*, *Saccharimonadales*, and *Saccharimonadi*) progressively increase starting from patients with mild cognitive decline to patients showing dementia. *Lactobacillus casei* has been associated with the regulation of synaptogenesis, synaptic refinement, and pruning in the hippocampus, which plays a key role on the cognitive functions [100,101,119]. It was demonstrated that treatment with a probiotic composition, including bifidobacteria (*Bifidobacterium longum*, *Bifidobacterium breve*, *Bifidobacterium infantis*), lactobacilli (*Lactobacillus acidophilus*, *Lactobacillus plantarum*, *Lactobacillus casei*, *Lactobacillus delbrueckii* subsp. *bulgaricus*), and *Streptococcus thermophilus*, enhanced neuroplasticity in an AD mouse model and reversed the deficit in long-term potentiation (LTP) [128]. Oral administration of a cocktail of antibiotics (ABX) containing kanamycin, gentamicin, colistin, metrodinazole, and vancomycin to the APP/PS1 mouse model of AD, starting at postnatal day 14 (P14) until P21, affects the production of both pro- and anti-inflammatory cytokines and Aβ plaque formation [129]. ABX treatment exclusively during P14 and P21 was able to reduce the Aβ plaque load in the hippocampus and cortex in 6.5-month-old male APP/PS1 mice [129]. Long- and short-term ABX treatment of male APP/PS1 mice reduced the number of Aβ plaque-associated microglia and astrocytes and modified microglial morphology [126]. Although ABX treatment is beneficial in AD mice models, ABX treatment cannot be employed in AD patients because the long-term administration of ABX will produce side effects. Moreover, it is possible that long-term ABX treatment is beneficial only in AD mice models, which exhibit a less complex composition of the gut microbiota when compared to humans and are subjected to a highly controlled diet and environment.

A clear demonstration of the role of microbiota in promoting AD was obtained by employing GF mice and fecal microbiota transplants [130]. Transplantation of fecal microbiota from an eight-month-old APPS21 mice model of AD to GF mice promoted an earlier AD pathology in GF mice [130].

Several studies demonstrated that the AD gut microbiota exhibits variations compared to healthy subjects and animal models. It seems that these variations in the AD gut microbiota induce modifications in the concentration levels of SCFAs, which play an anti-inflammatory function that modulates neuroinflammatory pathways [131]. In agreement, AD patients exhibit an imbalance in SCFAs production, which may contribute to AD progression. Propionate, a SCFA produced by gut bacteria, is increased in AD patients [132]. Moreover, SCFAs modulate Aβ aggregation.

Several studies indicate that PD progression is modulated by the microbiota. Interestingly, the first symptom of PD is constipation, which precedes the neurological symptoms [133]. In PD, a reduction in the SCFA-producing bacteria has been observed, which parallels the severity of cognitive and motor symptoms [134]. However, SCFA levels in the PD plasma are increased, potentially because of elevated intestinal permeability. Notably, transplantation of fecal microbiota derived from PD human donors in the Thy1-aSyn PD mice model deteriorated motor deficits more significantly than in Thy1-αSyn mice transplanted with fecal microbiota from healthy donors [135]. Moreover, opportunistic pathogens, including *Corynebacterium*, *Porphyromonas*, *Alistipes*, *Bacteroides*, *Escherichia*, *Megasphaera* and *Desulfovibrio*, are elevated in PD patients, while various bacteria, e.g., *Blautia*, *Coprococcus*, *Roseburia*, *Lachnospira*, the SCFA-producing *Fusicatenibacter* and *Faecalibacterium*, are decreased [136]. Interestingly, the reduction in SCFA-producing bacteria in PD patients correlated with the severity of cognitive and motor symptoms [137]. In addition, PD patients presented increased expression of several inflammatory markers in the colon and feces, including CCL2, CCL5, CCR5, IL-1β, IL-6, IL-8, IL-17A, IFN-β, IFN-γ, TNFα, TLR2, and TLR4, as well as increased numbers of CD3+ T cells in the colon, unveiling the presence of gut inflammation that may participate in the neurodegenerative processes in PD [138,139].

### 3.4. Role of the Enteric Nervous System (ENS) on the Microbiota–Gut–Brain Axis

The brain and gut belong to two different systems in function and physiology, and there are three ways to achieve communication between them: the neural pathway, the immune pathway and the neuroendocrine pathway [74].

Neurons connecting the CNS and the GI tract transmit inflammatory signals to obtain a physiological balance. The ENS is composed of neurons and glial cells located in the intestinal submucosa and intestinal muscularis propria. ENS exhibits an important role in the connection between gut microbiota and the brain. The vagus nerve (VN) acts as a bridge connecting the ENS and the CNS. Gastrointestinal physiology and function are controlled in a CNS-independent manner. However, GI can also communicate with the CNS through the VN, microbiota metabolites, and neuroendocrine pathways. VN, the principal component of the parasympathetic nervous system, is a mixed nerve composed of 80% afferent and 20% efferent fibers [140]. The sensory fields of gut vagal neuron afferences are equipped with receptors that sense inflammatory modulators in the intestinal microenvironment [141]. Microbiota-derived metabolites can stimulate the afferent neurons in the ENS, leading to modifications in electrical activity in its related brain regions through the VN. Vagal afferent fibers transmit the signal to the brain regions. The ENS exhibits a long-term bidirectional interaction with the microbiota. The ENS produces an excellent environment for the microbiota to play a crucial role in the growth, expansion, structural and functional integrity of the ENS. The alteration of gut microbiota may induce a dysfunction in the structure and function of both gut and ENS, followed by the loss of gut neurons in the submucosal and intramuscular plexus of the ileum and proximal colon [90]. Besides their role as a bridge communicating signals between the ENS and CNS, the neurons in the vagal ganglion can detect the mechanical distension of the GI tract, innervate the intestinal villi, sense nutrients, and control intestinal motility. These vagal-mediated GI hormone responses play an important role in the regulation of eating patterns and glucose homeostasis.

Central regulation of intestinal inflammation occurs through the vagal descending path from the dorsal motor nucleus of the vagus. A cholinergic anti-inflammatory pathway has been described through VN’s fibers that stimulate enteric neurons that inhibit macrophage release of inflammatory cytokines IL-1α, IL-6, IL-18, and TNF-α. Moreover, gut immune processes present in the intestine modulate neuroimmune homeostasis to protect the ENS. Colonization and translocation of pathogenic bacteria into the gut induce neuroinflammation, which in turn affects cognitive function. Infection may destroy the tight junction of the intestinal barrier, damaging the intestinal barrier function [142]. This damage, in turn, has an effect on the BBB permeability [143]. To remove bacteria, neurons present in the gut modulate the gut’s immune barrier through interleukin-18 (IL-18) [90]. Gut-derived cells may have a local action on the CNS immune cells. A subset of IFN-α–producing meningeal NK cells derived from the gut promotes the development of neuro-immune regulatory astrocytes that express LAMP1 and TRAIL [144].

The HPA axis exhibits an important role in maintaining the homeostasis of the gut–brain axis by acting as the communication pathway between the gut and the brain [74]. The HPA axis secretes several molecules that are essential for the regulation of sympathetic and parasympathetic, metabolic, immune, and CNS responses. The neuropeptide corticotropin-releasing factor (CRF), secreted by the paraventricular nucleus of the hypothalamus, is important for the homeostasis of the physiological equilibrium. The CRF stimulates the pituitary gland to synthesize the adrenocorticotropic hormone (ACTH), which controls the release of glucocorticoids (cortisol and corticosterone) from the adrenal cortex [145]. Cortisol in irritable bowel syndrome can activate immune cells and afferent fibers in the gastrointestinal tract [146]. Furthermore, CRF stimulates the locus coeruleus to produce catecholamines, modulating the noradrenergic activity of the brain [147]. The bidirectional interaction between gut microbiota and HPA is relevant for the maintenance of a healthy life [148]. On the other hand, stress-induced HPA response affects the intestinal barrier permeability, leading to dysbiosis [149,150].

As mentioned above, the brain communicates with the GI through the VN. Within the GI, there are different afferents of the VN that provide different signals to the brain. This bidirectional communication is influenced by the gut microbiota: signals from the brain modulate the gut microbiota composition via the VN and, in turn, by enteroendocrine cells, which secrete neurotransmitters that modulate the gut microbiota development and adaptation to the GI tract [151]. Five main enteroendocrine hormone types are secreted in the GI: gastric inhibitory peptide, ghrelin, 5-HT, somatostatin, and cholecystokinin (or neurotensin). Multiple hormones may be secreted by the gut epithelial cells. The gut synthesizes neurotransmitter substances like dopamine (DA), serotonin, and kynurenine through the 5-hydroxytryptophan (5-HT) pathway or the kynurenine pathway [152]. The 5-HT pathway exhibits an important role in modulating emotions, appetite, sleep, and other physiological functions. Almost all peripheral 5-HT intermediates derive from enterochromaffin cells (ECs), which are regulated by the gut microbiota and synthesize 5-HT from tryptophan [153]. The 5-HT released in the bloodstream is transported to defined brain regions, such as the cerebral cortex, hypothalamus and amygdala, in order to ensure specific physiological functions. Furthermore, 5-HT also influences physiological processes such as bowel movement and intestinal barrier permeability. Tyrosine metabolism produces neurotransmitters such as DA, norepinephrine (NE), and adrenaline in the gastrointestinal tract. Current studies reveal that the gut microbiota convert tyrosine into 4-ethylphenyl sulphate (4EPS), a neuroactive microbial molecule, which reduces the maturation of brain myelinated oligodendrocytes [151].

### 3.5. Microbiota–Gut–Brain Axis: Molecular Pathways, Mediators, and Microbiota Metabolites Involved

The bidirectional communication between the gut and the brain includes several mediators that have a crucial role in this interaction. Among these mediators, neurotransmitters like glutamine, dopamine, and GABA, which are produced in both the gut and the brain, microbiota-derived metabolites, including SCFAs and indoles, act as signaling molecules influencing brain function. Furthermore, immune system components, including cytokines and T-cells, can also modulate the activity of the gut–brain axis. 

Serotonin synthesis takes place in the enterochromaffin cells (EC) and enteric nerves. The serotonin secretion is regulated by the transient receptor potential (TRP) cation channel TRPA1 activity [154]. Serotonin receptors are divided into seven classes (5-HT_1_ to 5-HT_7_) and include a total of 14 known serotonin receptors [155]. 5-HT_1_ contains five subclasses (5-HT_1A_, 5-HT_1B_, 5-HT_1D_, 5-HT_1E,_ and 5-HT_1F_), 5-HT_2_ is divided in three subclasses (5-HT_2A_, 5-HT_2B_, and 5-HT_2C_), whereas 5-HT_5_ contains two subclasses (5-HT_5A_ and 5-HT_5B_) [156]. 5-HT_4_ activation prevents apoptosis in enteric neurons and induces ENS growth and maintenance [157]. The majority of serotonin secretion is present in the GI tract, while only 5% is present in the brain. Within the gut, about 90% of serotonin is in ECs and about 10% is in enteric neurons, pancreatic cells and mast cells. Cofactors, such as vitamin B6, vitamin B3, and magnesium, are essential for serotonin production. 5-HT produced from ECs enters the bloodstream, surrounding tissues and gut lumen. 5-HT is transported by serotonin reuptake transporter (SERT) into epithelial cells and platelets, then it is degraded to 5-hydroxyindoleacetic acid (5-HIAA). Five (5-HTR1, 5-HTR2, 5-HTR3, 5-HTR4, and 5-HTR7) of the seven 5-HT receptor (5-HTR) families are expressed in the gut smooth muscle, enteric neurons, enterocytes, and immune cells, mediating secretomotor and sensory functions such as nausea, vomiting, intestinal fluid and mucus secretion, and peristaltic movement [158]. In response to food intake, 5-HT plays an essential role in the peristaltic reflex creation, segmentation, and mucosal stimulation under normal and pathological conditions. Furthermore, 5-HT promotes the gastric mucus and fluid production to inhibit gastric acidity. Mucus acts as a physical barrier for microorganisms, diffusion of toxins, and as an antioxidant [159]. Serotonin receptors are expressed by innate immune cells and play a crucial role in the recruitment of these cells at the site of acute inflammation [160]. Serotonin exhibits modulating roles, acting as pro- or anti-inflammatory mediator: 5-HTR4 induces an anti-inflammatory effect and acts mostly at basal or normal conditions; on the contrary, 5-HTR7 mediates pro-inflammatory pathways, acting almost exclusively in pathological conditions [161]. 5-HT promotes angiogenesis during several physiological processes, such as organ development, reproduction, wound healing and pathological conditions like diabetic retinopathy, rheumatoid arthritis, age-related macular degeneration, and tumor growth and metastasis. Alteration of the serotonin-dependent pathways in the gastrointestinal system impairs brain functions, such as those involved in mood, sleep, and behavior [162,163]. 

About 50% of the synthesis of dopamine occurs in the gut. Dopamine induces a signal transduction through G protein-coupled receptors, named D receptors. Dopamine has a wide range of activities, playing an important role in cognition, reward, satiety, voluntary motor movements, pleasure, and motivation. It is essential for excitement, mobility, mood, and the execution of activities that involve fast decisions and learning. The correlation between the gut microbiota and dopamine function has been studied. Dopamine causes vasodilation (at moderate concentrations), stimulates sodium excretion and urine production by the kidneys, and suppresses intestinal movement and insulin secretion by the pancreas. Dopamine in the gut can influence gastrointestinal secretions, motility, and even protect against ulcers [164]. Microbiota exhibits neuroprotective effects by reducing dopamine depletion. The microorganisms of microbiota, such as *Bacillus mycoides*, *B. subtilis*, *Proteus vulgaris*, and *Serratia marcescens*, produce dopamine at micromolar concentrations. Dopamine at micromolar concentrations is also produced by *Morganella morganii* (2.46 mg/L, ~16 μM), *Klebsiella pneumonia* (1.06 mg/L; 6.9 μM) and *Hafnia alvei* (0.73 mg/L; 4.7 μM) [165]. Dopamine receptors are expressed on immune cells’ surface, including T and B lymphocytes, dendritic cells, macrophages, neutrophils, NK cells, and T-regulatory cells [166]. Dopamine’s anti-inflammatory role is associated with the suppression of macrophage activation. Indeed, dopamine inhibits the synthesis of the inflammation activator IL-12 in response to a bacterial antigen (LPS) and increases the production of anti-inflammatory factor IL-10, which in turn promotes the secretion of dopamine by the neurons of the tegmental area [167]. Dopamine can also suppress the function of pre-activated T cells [168].

On the other hand, dopamine stimulation of D_1_ receptors of the human T regulatory lymphocytes T_reg_s (CD4 + CD25high) causes inhibition of their immunosuppressive activity and a decrease in IL-10 and TGF-β production. By “suppressing immune-suppressors”, dopamine is expected to activate immune responses. In agreement, dopamine inhibits the second major component of the immunosuppression system: the myeloid-derived suppressor cells (MDSCs). Dopamine activates the D_1_ receptors of MDSCs, reducing their capability to inhibit T_reg_ proliferation and secretory function [169].

Neuroactive amino acids, including glutamic acid, aspartic acid, and α-aminobutyric acid (GABA), are present in the mammalian organism in two forms: free and in a complex. Several microorganisms synthesize aspartic and glutamic acid, GABA and glycine. GABA exhibits an important role in behavior, cognition, stress reduction, menopausal syndrome symptoms, immunity activation, decrement of depression and insomnia, regulation of blood pressure, counteraction of obesity, and improvement of visual cortex performance [170]. GABA is recognized by three receptors called (GABARs): alpha, beta, and gamma. The activation of these receptors induces ion channel opening at the inhibitory synapses, leading to the entrance of chloride ions into the cell and the exit of potassium ions. Glutamate decarboxylase, produced by bacteria, catalyzes the α-decarboxylation of l-glutamate to produce GABA [171]. GABA can also be produced from ornithine, arginine, and putrescine, and numerous human gut bacteria contain homologous enzymes involved in GABA biosynthesis. The Parabacteroides and *Eubacterium* genera produce GABA, along with *Lactobacillus*, *Bifidobacterium*, *Bacteroides*, *Blautia*, and in particular *Bacteroides fragilis* [172]. Fecal microbiota transplantation, containing Lactobacillus, enhances plasma GABA levels, ameliorating depression [173]. Conversely, reduced levels of *Bifidobacterium pseudolongum* and elevated levels of *Desulfovibrio piger* and *Mucispirillum schaedleri* in the microbiota reduce hippocampal GABA levels. *Lactobacillus rhamnosus* can modify GABARs expression, reducing depression and anxiety. GABA exhibits an effect on the GI tract, limiting its pain sensitivity [174]. An imbalance in the GI microbiota frequently results in reduced microbial GABA synthesis [172].

A large family of metabolites has been characterized, such as short-chain fatty acids (SCFAs), bile acids, and choline metabolites. These metabolites can modulate energy metabolism, nutrient absorption, and the regulation of gut microbiota composition [175].

Gut microbiota produces a large family of enzymes that transform a variety of compounds that are indigestible by human enzymes [176]. Thus, several metabolites with a wide spectrum of bioactivities will be generated. Three types of metabolites can be obtained: (i) metabolites generated by gut microbiota directly from diets, such as SCFAs and indole derivatives; (ii) metabolites obtained by the host and modified by gut microbiota, such as secondary bile acids; (iii) metabolites that are produced de novo, such as polysaccharide A. SCFA is the most studied metabolite. SCFAs are saturated aliphatic acids with a number of carbons that range from one to six. Acetate, propionate, and butyrate are the most common SCFAs produced by gut microbiota. Bacteria produce SCFAs, generated following fermentation of non-digestible carbohydrates, including large polysaccharides (resistant starches, cellulose, hemicellulose, pectins, and gums), playing a crucial role in maintaining gut health [177]. *Lactobacillus* and *Clostridium* (*Firmicutes*) are largely engaged in the fermentation of dietary fiber to yield SCFAs, principally acetate, propionate, and butyrate, which are crucial for regulating physiological processes. SCFAs are considered an important energy source for gut cells. They maintain the gut barrier function by supporting the integrity of the intestinal barrier, preventing harmful substances from leaking into the bloodstream. SCFAs can affect glucose and lipid metabolism, potentially influencing weight management and preventing metabolic disorders. The SCFAs are rapidly absorbed by colonocytes in the large intestine via hydrogen-dependent or sodium-dependent monocarboxylate transporters.

## 4. Polyphenols

### 4.1. General

Polyphenols are plant-derived bioactive compounds abundant in fruits, vegetables, tea, and cereals, and are increasingly recognized for their role in neuroprotection. In the context of dementia, polyphenols exert beneficial effects by modulating key pathogenic mechanisms, including oxidative stress, neuroinflammation, and synaptic dysfunction [178,179]. These compounds can also influence neuronal signaling and reduce the accumulation of toxic protein aggregates associated with Alzheimer’s and Parkinson’s diseases [180]. Importantly, despite their limited bioavailability, regular intake of polyphenol-rich foods has been associated with a lower risk of cognitive decline and dementia, partly through interactions with the gut microbiota and the modulation of the gut–brain axis [181].

Polyphenols are structurally diverse compounds characterized by one or more phenolic rings bearing hydroxyl groups, which determine their chemical reactivity and biological activities [182]. They are classified based on structural features such as the number of phenolic units, degree of polymerization, and carbon skeleton [183]. These compounds may occur in free or conjugated forms, influencing their solubility and bioavailability [184].

Polyphenols are synthesized through the shikimate and aceto-malonate pathways, with the shikimate–phenylpropanoid pathway being the primary route linking primary metabolism to aromatic amino acid and phenolic compound biosynthesis [185,186]. Phenylalanine acts as a key precursor, and the enzyme phenylalanine ammonia-lyase (PAL) catalyzes the first committed step in this process [187,188]. Polyphenol biosynthesis is tightly regulated by environmental factors such as light, nutrient availability, and stress conditions [189]. Structural diversity is further increased by post-biosynthetic modifications, including glycosylation and sequestration [190].

Polyphenols are divided into five main classes: flavonoids, phenolic acids, stilbenes, lignans, and tannins. Flavonoids, the most abundant group, share a C6–C3–C6 structure and include subclasses such as flavonols, flavones, and anthocyanins [191,192]. Phenolic acids are categorized into hydroxybenzoic and hydroxycinnamic acids, often present in conjugated forms, affecting their bioavailability [193,194]. Stilbenes, including resveratrol, are synthesized via the phenylpropanoid pathway and exhibit antioxidant and anti-inflammatory properties [195,196,197,198,199,200].

Lignans are diphenolic compounds derived from phenylpropanoid dimers and serve as precursors of bioactive enterolignans produced by gut microbiota, contributing to antioxidant and cardioprotective effects [201,202,203]. Tannins, classified into hydrolyzable and condensed forms, are high-molecular-weight polyphenols with strong protein-binding capacity and diverse biological activities, including antioxidant and antimicrobial effects [204,205,206,207].

Dietary polyphenols are widely distributed in plant-based foods and beverages. High concentrations are found in spices, cocoa, berries, nuts, and vegetables, while tea, coffee, and red wine represent major beverage sources [208,209,210]. Their content and bioavailability depend on plant origin, environmental conditions, and food processing methods.

Gut microbial metabolism plays a critical role in determining the biological activity of dietary polyphenols. A large proportion of ingested polyphenols reaches the colon in a poorly absorbed form, where they are extensively metabolized by the gut microbiota through enzymatic reactions such as deglycosylation (mediated by β-glucosidases), dehydroxylation, demethylation, and ring fission [176,211]. These microbial transformations generate low-molecular-weight metabolites, including phenylpropionic acids, phenylacetic acids, benzoic acids, and urolithins, which generally exhibit higher bioavailability compared to their parent compounds [212].

Notably, specific microbial taxa such as *Eggerthella lenta*, *Gordonibacter urolithinfaciens*, and *Bifidobacterium* spp. are involved in these biotransformation processes, highlighting the importance of interindividual microbiota composition in determining metabolic outputs [213]. Importantly, several of these metabolites are able to cross the blood–brain barrier and exert biological effects at the central nervous system level [178].

These microbiota-derived metabolites have been shown to exert neuroprotective effects through multiple mechanisms, including the reduction in oxidative stress, modulation of neuroinflammatory signaling pathways (e.g., NF-κB and Nrf2), and regulation of neuronal survival and synaptic plasticity [214]. Therefore, the health effects of dietary polyphenols are largely dependent on their microbial biotransformation, emphasizing the concept that polyphenols act as precursors of bioactive metabolites rather than as bioactive compounds per se.

### 4.2. Interest for General Health

To better understand the role of polyphenols on human health and disease prevention, it is essential to characterize the main dietary sources of each polyphenol. Polyphenols are typically ingested as complex mixtures, with their biological effects largely dependent on interactions with other dietary components and the gut microbiota [178]. The Mediterranean diet (MeDi), which exhibits high polyphenol content, exhibits lower cardiovascular mortality, strongly suggesting that polyphenols are health-promoting bioactive compounds. Several studies show that consumption of polyphenol-rich foods is associated with a reduced risk of chronic diseases and improved cardiometabolic health. In all-cause mortality, individuals consuming polyphenol-rich diets exhibit approximately a 7% lower risk of death compared with those with lower polyphenol intake [215]. Moreover, high polyphenol intake correlates with enhanced immune protection [182]. Polyphenols exhibit high antioxidant effects by scavenging reactive oxygen species and modifying oxidative stress, thereby potentially protecting against oxidative stress-related conditions such as cardiovascular disease, diabetes, and neurodegenerative disorders [216]. Polyphenols can interact with gut microbiota, which in turn accelerates the metabolism of polyphenols to bioactive metabolites, reducing inflammation and modulating human metabolism [217]. Polyphenol-induced mechanisms of action have been extensively investigated across in vitro experiments, animal models, and human studies. Evidence consistently indicates that regular consumption of polyphenol-rich foods is associated with quantifiable decreases in all-cause mortality risk, improved cardiometabolic markers, and a lower prevalence of metabolic disorders, reinforcing the importance of plant-based dietary patterns [215]. Polyphenols exert potent antioxidant effects by directly scavenging reactive oxygen species and modulating oxidative stress pathways, offering potential protection against related conditions such as cardiovascular disease and neurodegenerative disorders [216]. A growing body of evidence from systematic reviews and meta-analyses substantiates the beneficial impact of polyphenols on cardiometabolic function. High dietary intake or supplementation with polyphenols is associated with improvements in key disease risk factors, including reduced blood pressure, favorable lipid profile changes, and enhanced glucose homeostasis [218]. Recent large-scale meta-analyses quantified these effects; for instance, catechin intake has been shown to reduce systolic and diastolic blood pressure by approximately 1.56 mmHg and 0.95 mmHg, respectively, while anthocyanins improve lipid profiles, and curcumin enhances glucose metabolism [218]. Higher overall polyphenol consumption is associated with an approximately 22% lower prevalence of metabolic syndrome, and a similar association has been observed for flavonoids, flavan-3-ols, and stilbenes. Clinical cohort studies report an inverse correlation between dietary intake of lignans and stilbenes and hypertension risk. Taken together, these quantitative and mechanistic findings provide strong support for dietary polyphenols as key contributors to long-term health. Collectively, the evidence underscores their role in disease prevention and health maintenance through interconnected pathways involving antioxidant activity, modulation of inflammatory and metabolic processes, and beneficial interactions with the gut microbiota.

### 4.3. Role on Brain Health

While genetic predisposition, environmental exposures, and lifestyle factors are established contributors, growing evidence indicates that dietary components particularly plant-derived phytochemicals play a pivotal role in preserving cognitive function and delaying the onset of neurodegenerative diseases [73]. The neuroprotective effects of polyphenols are mediated through multiple mechanisms, including the reduction in oxidative stress, the modulation of neuronal signaling and neurotrophic factors, the inhibition of pathological protein aggregation that is a characteristic of several diseases like Alzheimer’s, and the direct support of cognitive performance [219]. Oxidative stress, driven by an imbalance in reactive oxygen species (ROS), is a key contributor to neuronal damage. Polyphenols counteract oxidative damage through a potent free radical–scavenging capability, thereby helping to maintain redox balance in the brain. Polyphenols act as antioxidants by donating electrons or hydrogen atoms to neutralize harmful ROS such as superoxide and hydroxyl radicals [220]. Specific flavonoids like quercetin, characterized by hydroxyl groups in their structure, are particularly effective in directly scavenging free radicals, thereby alleviating oxidative stress in neuronal cells [220]. Beyond direct scavenging, polyphenols enhance endogenous antioxidant defenses. They modulate critical signaling pathways, including the activation of the transcription factor Nrf2, which upregulates protective enzymes like superoxide dismutase (SOD) and glutathione peroxidase (GPx) [221]. Moreover, polyphenols can inhibit ROS-generating enzymes like NADPH oxidase (NOX) and modulate pathways involving MAPK and NF-κB, which are central to inflammatory responses [222]. They also upregulate brain-derived neurotrophic factor (BDNF), a key protein for synaptic plasticity and memory [223]. Clinical data from randomized controlled trials substantiates these mechanistic insights. Recent studies demonstrate that supplementation with polyphenol-rich nutraceuticals leads to significant improvements in cognitive function and increases in neuroplasticity-related biomarkers, such as BDNF and CREB, in adults compared to placebo groups [223]. These results underline the potential of polyphenol-based therapy to reinforce synaptic communication and produce protection against age-related cognitive decline. Several studies reveal that polyphenols can reduce neuroinflammation, attenuate Aβ aggregation, and modulate microglial activation [220]. Taken together, these findings suggest that dietary polyphenols contribute to brain health through multiple interrelated biological mechanisms, including antioxidant defense, anti-inflammatory modulation, neurotrophic support, and potential effects on vascular and metabolic factors, supporting their inclusion as a part of dietary strategies aimed at preserving cognitive function and reducing the risk of neurodegenerative disease.

### 4.4. Polyphenols, Gut Microbiota Metabolism, and Molecular Mechanisms in Dementia

The majority of dietary polyphenols (approximately 90–95%) escape absorption in the small intestine and reach the colon largely intact, where they interact extensively with the gut microbiota [224,225]. In this context, the gut microbiota acts as a metabolic “secondary organ,” converting complex polyphenolic structures into smaller, more bioavailable and biologically active metabolites through enzymatic reactions, including deglycosylation, ring fission, dehydroxylation, reduction, and demethylation [226,227]. These transformations generate a wide range of low-molecular-weight compounds such as phenolic acids, urolithins, phenyl-γ-valerolactones, and enterolignans, which exhibit enhanced systemic bioavailability and biological activity compared with their parent compounds [226].

This metabolic interplay is fundamentally bidirectional: while gut microbiota transforms polyphenols into active metabolites, polyphenols simultaneously modulate microbial composition and function by acting as selective substrates for specific bacterial taxa. Polyphenol intake promotes the growth of beneficial commensal bacteria, including *Bifidobacterium*, *Lactobacillus*, *Akkermansia muciniphila*, and *Faecalibacterium prausnitzii*, while suppressing opportunistic pathogens such as *Clostridium perfringens*, *Escherichia coli*, and *Helicobacter pylori* [217,228]. This remodeling of the gut microbiota contributes to improved microbial diversity, enhanced metabolic activity, and restoration of intestinal homeostasis.

A key functional outcome of polyphenol-driven microbiota remodeling is the increased production of SCFAs, particularly acetate, propionate, and butyrate, which act as critical mediators of gut–brain axis signaling, contributing to neuroprotection by modulating neuroinflammation, enhancing neurogenesis, and preserving BBB integrity [229,230]. Notably, microbial metabolism of polyphenols varies among individuals, leading to distinct “metabotypes” that influence both microbial composition and host physiological responses [231,232].

Following absorption, polyphenol-derived metabolites undergo extensive phase II metabolism in enterocytes and hepatocytes, including glucuronidation, sulfation, and methylation, which regulate their solubility, systemic distribution, and biological activity [233]. These processes, together with dietary factors such as food matrix, lipid co-consumption, and processing conditions, critically determine polyphenol bioavailability and metabolic fate [234]. This supports the emerging concept that polyphenols act as prodrugs, whose biological effects are primarily mediated by microbiota-derived and host-conjugated metabolites rather than by the native compounds themselves.

At the molecular level, these metabolites function as potent signaling molecules regulating key pathways involved in neurodegeneration. Central among these are the nuclear factor erythroid 2-related factor 2 (Nrf2) and nuclear factor-κB (NF-κB) pathways [235]. For example, urolithin A activates Nrf2-dependent antioxidant responses, inducing cytoprotective enzymes such as heme oxygenase-1 (HO-1) and NAD(P)H quinone dehydrogenase 1 (NQO1), while phenolic acids inhibit NF-κB signaling, reducing the expression of pro-inflammatory mediators including IL-6, TNF-α, and inducible nitric oxide synthase (iNOS) [220].

Beyond redox and inflammatory pathways, polyphenol-derived metabolites also exert epigenetic regulation by modulating DNA methyltransferases (DNMTs) and histone deacetylases (HDACs), thereby altering gene expression patterns associated with inflammation and cellular stress responses [236]. Additionally, these metabolites act as ligands for key receptors such as the aryl hydrocarbon receptor (AhR) and peroxisome proliferator-activated receptor-γ (PPARγ), influencing immune tolerance, epithelial barrier function, and metabolic regulation [237].

Importantly, these molecular and metabolic effects extend beyond the gut, as circulating microbiota-derived metabolites can cross the blood–brain barrier and modulate neuronal and glial cell function, thereby influencing neuroinflammation, synaptic plasticity, and cognitive processes through the gut–microbiota–brain axis [148]. Collectively, this integrated host–microbiome interaction positions polyphenols as key modulators of cellular resilience and neuroprotection in dementia, with their efficacy largely dependent on individual microbiota composition and metabolic capacity.

### 4.5. Neuron–Glia–Vascular Interactions and Modulation by Polyphenols

The neuron–glia–vascular unit (NVU), comprising neurons, astrocytes, microglia, endothelial cells, and pericytes, represents a critical structural and functional interface for maintaining brain homeostasis and cognitive function. In neurodegenerative diseases and dementia, this tightly regulated system becomes disrupted, leading to BBB breakdown, impaired cerebral blood flow, and exacerbated neuroinflammation. Endothelial dysfunction and pericyte loss contribute to increased BBB permeability, facilitating the infiltration of peripheral inflammatory mediators and neurotoxic molecules into the brain parenchyma, thereby accelerating neuronal injury and cognitive decline [112,143]. In this context, the NVU constitutes a primary target of neurodegenerative processes and a key therapeutic focus.

Emerging evidence indicates that dietary polyphenols exert neuroprotective effects by modulating this neuron–glia–vascular axis at multiple levels. In neurons, polyphenols reduce amyloid-β and α-synuclein aggregation, enhance autophagy and mitophagy, and promote synaptic plasticity through activation of BDNF/CREB signaling pathways, thereby supporting neuronal survival and cognitive function [219,223]. At the glial level, polyphenols regulate microglial activation by suppressing pro-inflammatory M1 polarization induced by amyloid plaques and α-synuclein aggregates. This effect is mediated through inhibition of NF-κB signaling, TLR4 activation, and NLRP3 inflammasome assembly, resulting in reduced production of pro-inflammatory cytokines such as IL-1β, TNF-α, and IL-6, while promoting an anti-inflammatory M2 phenotype associated with phagocytosis and tissue repair [50,220]. Notably, curcumin inhibits NLRP3 inflammasome activation and IL-1β release in Alzheimer’s disease models, while quercetin reduces TNF-α and IL-6 secretion in activated microglia.

Astrocytes also represent key targets of polyphenol action. Compounds such as resveratrol and epigallocatechin-3-gallate (EGCG) attenuate reactive astrogliosis, preserve glutamate transporter expression (GLT-1/EAAT2), and reduce the release of complement component C3 associated with the neurotoxic A1 phenotype. These effects restore astrocytic functions, including glutamate clearance, metabolic support, and neurotrophic factor production, particularly brain-derived neurotrophic factor (BDNF), thereby enhancing synaptic plasticity and neuronal resilience [48].

At the vascular level, polyphenols improve cerebrovascular function and BBB integrity. Flavanols and anthocyanins enhance nitric oxide (NO) bioavailability via endothelial nitric oxide synthase (eNOS) activation, reduce oxidative stress–mediated NO degradation, and restore endothelial-dependent vasodilation, contributing to improved neurovascular coupling observed in clinical studies [238,239]. In parallel, polyphenols upregulate tight junction proteins such as claudin-5, occludin, and ZO-1, thereby reinforcing BBB integrity and limiting paracellular permeability [48,112].

Importantly, these effects are further amplified by microbiota-derived metabolites of polyphenols, including SCFAs and urolithins, cross the BBB and modulate NVU function, contributing to the maintenance of CNS homeostasis.

Collectively, these findings demonstrate that polyphenols exert a multi-target neuroprotective action by restoring the functional integrity of the neuron–glia–vascular unit. This integrated mechanism highlights the central role of polyphenols as modulators of neurodegenerative pathways in dementia and establishes a mechanistic link between dietary polyphenols, gut microbiota metabolism, and central nervous system resilience.

## 5. Effects of Polyphenols on the Cognitive Functions: Preclinical Studies

### In Vitro Studies and Animal Models

The brain is particularly vulnerable to oxidative stress due to its high oxygen consumption, abundance of polyunsaturated fatty acids, and relatively limited antioxidant defenses. Mitochondrial dysfunction, excessive production of reactive oxygen species (ROS), and impaired DNA repair systems contribute significantly to the progression of neurodegenerative diseases, including Alzheimer’s disease (AD) and Parkinson’s disease (PD). In this context, dietary polyphenols have emerged as promising neuroprotective agents, not only due to their intrinsic antioxidant properties but also through their ability to modulate the gut microbiota and generate bioactive metabolites that target central nervous system (CNS) pathways.

In the colon, dietary polyphenols undergo extensive biotransformation by the gut microbiota [224,225]. Through enzymatic reactions such as deglycosylation, dehydroxylation, demethylation, and C-ring cleavage, complex polyphenols are converted into low-molecular-weight metabolites, including phenolic acids, urolithins, equol, and phenyl-γ-valerolactones [226,240]. These transformations involve specific bacterial taxa such as *Bacteroides*, *Bifidobacterium*, *Lactobacillus*, *Eggerthella*, and *Gordonibacter*, and occur through cooperative metabolic processes (cross-feeding), which contribute to inter-individual variability in polyphenol bioactivity [241].

These microbiota-derived metabolites exhibit enhanced bioavailability and can cross the blood–brain barrier (BBB) via passive diffusion or monocarboxylate transporters, allowing them to directly influence CNS function [178]. Once in the brain, they exert multiple neuroprotective effects targeting key mechanisms of neurodegeneration. Notably, they activate antioxidant pathways such as Nrf2/ARE, leading to upregulation of endogenous defense systems including superoxide dismutase (SOD), catalase, and heme oxygenase-1 (HO-1), thereby reducing oxidative stress a central driver of neuronal damage. In parallel, they suppress neuroinflammatory signaling by inhibiting NF-κB activation and NLRP3 inflammasome assembly in microglia, resulting in decreased production of pro-inflammatory cytokines such as IL-1β, TNF-α, and IL-6 [242].

In addition to redox and inflammatory pathways, polyphenol-derived metabolites regulate neuronal survival and synaptic plasticity. They enhance the expression of brain-derived neurotrophic factor (BDNF) through activation of CREB signaling, promoting hippocampal neurogenesis, synaptic remodeling, and long-term potentiation processes essential for learning and memory. Furthermore, metabolites such as urolithin A induce mitophagy, facilitating the clearance of dysfunctional mitochondria and interrupting the cycle of oxidative stress and neuroinflammation [243]. These mechanisms are particularly relevant in neurodegenerative disorders characterized by mitochondrial dysfunction and synaptic loss.

The gut microbiota also plays a central role in shaping the functional output of polyphenols by modulating their bioavailability and metabolic fate. Only a small fraction (~10%) of polyphenols is absorbed in the small intestine, where they undergo phase I and phase II metabolism, including glucuronidation, sulfation, and methylation [225]. The remaining fraction is metabolized in the colon, generating bioactive compounds that act as the primary mediators of systemic and CNS effects. This supports the concept that polyphenols function as microbiota-dependent prodrugs, whose biological activity depends on individual microbial composition.

Polyphenols also exert reciprocal effects on gut microbiota composition, promoting beneficial bacteria such as *Lactobacillus*, *Bifidobacterium*, *Akkermansia muciniphila*, and *Faecalibacterium prausnitzii*, while inhibiting pathogenic species including *Clostridium perfringens* and *Escherichia coli* [228,244]. These changes enhance microbial diversity and stimulate the production of SCFAs, particularly butyrate, propionate, and acetate, which play a critical role in gut–brain communication.

Importantly, gut dysbiosis is increasingly recognized as a contributing factor in neurodegeneration. Reduced microbial diversity and decreased SCFA production are associated with increased intestinal permeability, systemic inflammation, and exacerbation of neuroinflammatory processes, ultimately leading to cognitive impairment [245]. Polyphenols counteract these alterations by restoring microbial balance and enhancing the production of neuroprotective metabolites.

Specific polyphenols such as resveratrol, curcumin, quercetin, and fisetin have demonstrated robust neuroprotective effects in preclinical models of AD and PD. These compounds modulate multiple CNS pathways, including reduction in amyloid-β and α-synuclein aggregation, inhibition of neuroinflammation, enhancement of neurogenesis, and restoration of mitochondrial function [246,247,248]. For instance, resveratrol activates the SIRT1 and Nrf2/HO-1 pathways, reduces oxidative stress, and modulates microglial activation via CX3CL1/CX3CR1 signaling, while curcumin exerts anti-inflammatory and anti-amyloidogenic effects in multiple dementia models.

Collectively, these findings demonstrate that the neuroprotective effects of polyphenols are mediated through a complex, microbiota-dependent network involving metabolic transformation, immune modulation, and direct CNS signaling. This integrated framework highlights the central role of the gut–microbiota–brain axis in linking dietary polyphenols to neurodegenerative disease modulation and cognitive function preservation.

## 6. Effect of Polyphenols on Cognitive Function and Dementia: Clinical Studies

### 6.1. Clinical Trials and Dietary Interventions

Studies exploring the potential of polyphenols on cognitive function and dementia are not limited to preclinical trials. Various studies also reporting human clinical trials investigated the effects of different types of polyphenol-rich foods, extracts, and dietary patterns on overall cognitive performance and neurodegeneration [249]. To contextualize their clinical relevance, numerous randomized controlled trials (RCTs) and dietary intervention studies have evaluated the impact of polyphenols on cognitive outcomes across diverse populations, including healthy older adults, individuals with mild cognitive impairment (MCI), and people in the early stages of Alzheimer’s disease (AD). While these studies vary in design, polyphenol source, dosage, and cognitive endpoints, they collectively offer valuable insights about the potential of polyphenols to influence memory, executive function, and other cognitive domains. Table 1 summarizes key findings from representative human clinical trials, including intervention types, population characteristics, and primary cognitive outcomes. Additionally, it also reflects the diversity of polyphenol types studied (flavanols, anthocyanins, stilbenes, and curcuminoids), demonstrating how different classes of polyphenols may exert distinct effects on specific cognitive functions.

Overall, the clinical evidence underscores the potential of polyphenols as promising dietary agents in supporting cognitive health and mitigating age-related cognitive decline, particularly in populations with MCI and early-stage AD. RCTs across diverse polyphenol subclasses such as flavanols, anthocyanins, stilbenoids, and curcuminoids, have demonstrated significant benefits across multiple cognitive domains including memory, executive function, attention, and processing speed. Among the subclasses, cocoa flavanols have shown the most reproducible cognitive benefits, particularly in MCI. In general, high-dose flavanol interventions (≥500 mg/day) are associated with enhanced performance on executive function and processing speed tasks such as the Trail Making Test (TMT) A and B, verbal fluency, and global cognitive composites. For instance, Desideri and colleagues and Mastroiacovo and colleagues reported that eight weeks of 990–993 mg/day flavanol supplementation in older adults led to significantly better cognitive outcomes compared to low-dose controls [250,251]. Similarly, Sorond and colleagues and Brickman and colleagues found cognitive benefits linked to increased cerebral blood volume and improved neurovascular coupling, suggesting that vascular mechanisms may underpin these effects [238,239].

Anthocyanin-rich interventions, particularly from blueberries and grapes, have also demonstrated efficacy in MCI. For instance, Wood and colleagues as well as Whyte and colleagues reported that 12–13 weeks of wild blueberry extract supplementation in healthy older adults significantly improved immediate verbal recall and task-switching accuracy, and strongly enhanced episodic memory [252,253]. Although Miller and colleagues as well as Krikorian and colleagues used different sources of anthocyanin (Strawberries and grape juice), they also observed improvements in various cognitive domains, including paired associate learning and verbal recall [254,255]. Despite the fact that a study from Boespflug and colleagues did not observe behavioral improvements in MCI, the same report presented scans from functional magnetic resonance imaging (fMRI) that revealed enhanced parietal and occipital activation, suggesting anthocyanins may augment neural efficiency even without overt cognitive change [256].

**Table 1:** Synthesis of representative human clinical trials on polyphenols and cognition.

Polyphenol Type	Study Design	Participants (Condition/Age)	Population (N)	Intervention (mg/mL)	Duration of the Dose	Key Cognitive Outcomes Measured	Key Findings	References
Flavanols (Cocoa)	RCT ^1^, DB ^2^	Elderly with MCI ^3^ (mean age 71 y)	90	High-flavanol (990 mg), intermediate (520 mg), or low (45 mg) cocoa drink	8 weeks	^4^ TMT A and B, ^5^ MMSE, ^6^ VFT	High and intermediate flavanol groups showed significant improvements in TMT A and B, VFT, and insulin resistance. MMSE scores improved in the high-flavanol group.	Desideri et al. (2012) [250]
	RCT, DB	Healthy elderly (mean age 73 y)	90	High-flavanol (993 mg), intermediate (520 mg), or low (48 mg) cocoa drink	8 weeks	Composite cognitive score (TMT, VFT, MMSE, visual search)	High-flavanol group showed significantly improved composite cognitive score, particularly in tasks related to attention and executive function.	Mastroiacovo et al. (2015) [251]
	RCT, DB	Elderly with vascular risk factors (mean age 73 y)	60	High-flavanol (609 mg), low (13 mg) cocoa drink	4 weeks	MMSE, TMT (A–B)	Improvements in neurovascular coupling and TMT B performance for the high flavanols group	Sorond et al. (2013) [238]
	RCT, DB	Healthy elderly (Mean age 60 y)	41	High-flavanol (900 mg), low (45 mg) cocoa	12 weeks	Modified Benton Task (dentate gyrus-dependent memory task)	Increased cerebral blood volume and better performance in the dentate gyrus and in the case of high flavanol group	Brickman et al. (2014) [239]
	RCT	Healthy elderly (mean age 68.3 y)	40	High-flavanol (494 mg), low (23 mg) cocoa drink	4 weeks	Episodic memory, working memory, spatial memory, implicit memory, attention, and processing speed	Improvements in global cognition scores among the high flavanol group	Neshatdoust et al. (2016) [257]
Anthocyanins (Berries/Fruits)	RCT, DB	Healthy elderly (mean age 67.5 y)	37	460.8 mg of freeze-dried Blueberries	12 weeks	^7^ CVLT, Task-switching test	Improvements in executive function	Miller et al. (2018) [258]
	RCT, ^8^ PC	Healthy elderly (mean age 72.5 y)	61	302 mg of wild blueberry powder	12 weeks	^9^ RAVLT, Task-switching test	Improved immediate verbal recall and task-switching accuracy, compared to placebo	Wood et al. (2023) [252]
	RCT, PC	Healthy elderly (mean age 72.5 y)	122	500–1000 mg whole powder or 100 mg extract of blueberry extracts	13 weeks	RAVLT	Improved episodic memory performance, along with modest blood pressure reduction	Whyte et al. (2018) [253]
	RCT, DB, PC	Elderly with MCI (mean age 80 y)	16	24 g of freeze-dried blueberry powder	16 weeks	Task-switching, Stroop, ^10^ WCST	No significant group differences in cognitive performance, but ^11^ fMRI showed increased activation in parietal and occipital regions during cognitive tasks in the blueberry group, suggesting compensatory recruitment.	Boespflug et al. (2018) [256]
	RCT, DB, PC	Healthy elderly (mean age 67.5 y)	37	24 g of freeze-dried Strawberries	12 weeks	TMT, CVLT	Increased word recognition in the CVLT	Miller et al. (2021) [254]
	RCT, DB, PC	Elderly with MCI (mean age 80 y)	12	45 mL of concord grape juice	12 weeks	CVLT, spatial paired-associate learning	Improvements in verbal learning	Krikorian et al. (2010) [255]
Stilbenoid (Resveratrol)	RCT	Overweight elderly (mean age 79 y)	32	1000 mg/day of resveratrol	13 weeks	TMT	Improved psychomotor processing speed	Anton et al. (2018) [259]
	RCT, Crossover	Healthy elderly (mean age 24.8 y)	22	250–500 mg acute dose of trans-resveratrol	90 min	Serial subtraction tasks, rapid visual information processing	Dose-dependent increase in cerebral blood flow during task performance, measured by ^12^ fNIRS. No direct improvement in task performance.	Wightman et al. (2014) [260]
	RCT, PC	Elderly with mild to moderate AD (>49 years)	119	500 mg of resveratrol	13–52 weeks	^13^ CSF and plasma biomarkers (^14^ Aβ40, Aβ42, ^15^ τ), MMSE, ^16^ ADCS-ADL	The Resveratrol group showed less decline in CSF Aβ42 and plasma Aβ40 levels compared to placebo, suggesting target engagement. No significant effect on cognitive or functional outcomes.	Turner et al. (2015) [261]
Polyphenol extract (Curcumin)	RCT, DB	Non-demented adults (mean age 70 y)	40	90 mg of curcumin twice daily	78 weeks	^17^ FDDNP-PET (amyloid and τ), Buschke Selective Reminding Test, Brief Visual Memory Test	^17^ FDDNP-PET findings suggest that symptom benefits are associated with decreases in amyloid and tau accumulation in brain regions modulating mood and memory. Daily oral Theracurmin may lead to improved memory and attention in non-demented adults.	Small et al. (2018) [262]
	RCT, DB, PC	Healthy elderly (mean age 72.5 y)	60	400 mg Longvida^®^ (Curcumin extract)	4 weeks	Computerized Mental Performance Assessment System	Single-dose improved performance on working memory and sustained attention.	Cox et al. (2015) [263]
Polyphenoic-rich dietary patterns (Mediterranean Diet)	RCT	Healthy elderly (mean age 66.9 y)	447	^18^ MedDiet—olive oil, nuts, or low-fat control diet	Median 4.1 years	MMSE, Clock Drawing Test, Composite cognitive score	^18^ MedDiet groups showed significantly better cognitive function (global cognition, memory, executive function) compared to the control group.	Valls-Pedret et al. (2015) [264]

^1^ RCT—randomized controlled trials, ^2^ DB—double blind, ^3^ MCI—mild cognitive impairment, ^4^ TMT—trail making test, ^5^ MMSE—mini-mental state examination, ^6^ VFT—verbal fluency test, ^7^ CVLT—California verbal learning test, ^8^ PC—placebo, ^9^ RAVLT—Rey auditory verbal learning test, ^10^ WCST—Wisconsin card sorting test, ^11^ fMRI—functional magnetic resonance imaging, ^12^ fNIRS—functional near-infrared spectroscopy, ^13^ CSF—cerebrospinal fluid, ^14^ Aβ—amyloid-beta, ^15^ τ—Tau, ^16^ ADCS-ADL—Alzheimer’s disease cooperative study—activities of daily living, ^17^ FDDNP-PET—positron emission tomography, ^18^ MedDiet—Mediterranean diet.

Regarding Stilbenoids (resveratrol), the studies have reported contradictory results, including some promising outcomes in early-stage AD and MCI following treatment with polyphenols. Anton and colleagues reported improved psychomotor speed in overweight older adults at a dose of 1000 mg/day resveratrol over 12 weeks [259]. Although Turner and colleagues did not observe cognitive improvements in mild-to-moderate AD, in the same study, resveratrol preserved hippocampal volume and modified amyloid biomarkers (Aβ42) levels, suggesting the disease-modifying potential of resveratrol despite limited symptomatic change [261].

Moving on to curcuminoids, various reports from the literature have suggested measurable effects primarily in non-demented aging populations, but also showed early promise in MCI. Small and colleagues demonstrated that 18 months of Theracurmin^®^ supplementation (180 mg/day) in older adults with memory complaints, significantly improved verbal and visual memory and attention, with concurrent reductions in amyloid and tau deposition [262]. Cox and colleagues further observed acute improvements in attention and working memory post-curcumin ingestion, suggesting that both short- and long-term cognitive modulation may be feasible [263]. The gut microbiota-dependent mechanisms underlying these cognitive benefits, including curcumin biotransformation into neuroactive metabolites and microbiome reshaping, are discussed in detail in Section 6.6.

Lastly, studies from multi-compound polyphenol interventions and dietary patterns rich in polyphenols have reported benefits in those with cognitive vulnerability. For instance, Bensalem and colleagues found that a combined grape and blueberry extract improved verbal episodic memory specifically in participants with lower baseline cognitive performance, with gains linked to individual differences in metabolite production [265]. These data suggest that bioavailability of polyphenols and metabolic responsiveness may moderate cognitive outcomes. In agreement, the PREDIMED trial demonstrated that adherence to a Mediterranean diet rich in polyphenols was associated with sustained improvements in global and domain-specific cognitive function over a 4.1-year follow-up, emphasizing the long-term relevance of polyphenol-enriched diets [264].

Despite compelling research outcomes, several limitations complicate the interpretation and comparison of findings across studies. Considerable variability exists in study designs, including differences in sample sizes, intervention durations, cognitive assessment tools, and the specific dosing and sources of polyphenols. These methodological inconsistencies limit the ability to draw unified conclusions. Furthermore, individual responses to polyphenol interventions appear to be influenced by a range of factors, including genetic variation, baseline cognitive status, and particularly differences in polyphenol metabolism [266]. Among these, growing evidence suggests that variability in gut microbiota composition may play a crucial role in shaping the cognitive effects of polyphenols. Since many polyphenols have inherently low bioavailability, their bioactivity often depends on transformation by gut microbial enzymes into smaller, more absorbable metabolites [212,242]. Polyphenols also exert modulatory effects on the gut environment, influencing microbial diversity and selectively enriching beneficial taxa such as *Bifidobacterium* spp. and *Akkermansia* spp., while suppressing opportunistic pathogens [267,268]. This complex, bidirectional relationship suggests that the gut microbiota may not merely be a passive participant but an active mediator of polyphenol efficacy and its downstream effects on cognitive function.

### 6.2. Impact of Polyphenol-Rich Diets on Gut Microbiota and Cognitive Function

Polyphenols are increasingly recognized to influence cognitive health not only through direct antioxidant and anti-inflammatory actions, but also via their interactions with the gut microbiota, which is commonly termed as the microbiota–gut–brain axis. Many dietary polyphenols are poorly absorbed in the small intestine and substantial portions reach the colon, where they are metabolized by gut microbes into bioactive compounds. These microbiota-derived metabolites (e.g., small phenolic acids like phenyl-γ-valerolactones from cocoa, urolithins from ellagitannins, or dihydroresveratrol from resveratrol) can enter circulation and even cross the blood–brain barrier, potentially mediating neuroprotective effects [269,270,271]. Beneficial gut metabolites such as short-chain fatty acids (SCFAs) are also produced from polyphenol-rich diets. For instance, fermentation of dietary fiber and polyphenols by certain gut bacteria (e.g., *Roseburia* spp.) increases butyrate, an SCFA that acts as a histone deacetylase inhibitor with anti-inflammatory and memory-enhancing effects in rodent models [272]. Moreover, polyphenol interventions often modulate systemic biomarkers of brain health, for example, increasing neurotrophic factors and improving cerebrovascular function, highlighting a complex interplay between microbiota metabolism and cognitive outcomes [273]. In this perspective, below, we discuss how specific classes of polyphenols (flavanols, anthocyanins, stilbenes, curcuminoids) and polyphenol-rich dietary patterns influence gut microbiota composition and function, and how these changes correlate with cognitive modulation, including key biomarkers.

### 6.3. Flavanols and the Gut–Brain Axis

Flavanols (flavan-3-ols) from cocoa, tea, and certain fruits are known for cognitive benefits, especially in aging populations. The pivotal aspect of their action is their prebiotic-like effect on the gut microbiota. Cocoa flavanols are minimally absorbed in the upper gut and the majority reach the colon, where resident microbes metabolize them into smaller phenolics such as phenyl-γ-valerolactone and phenylvaleric acids. This microbial biotransformation not only increases flavanol bioavailability but also promotes beneficial gut bacteria. Studies have noted that cocoa and other flavanol-rich foods enrich bacterial genera like *Akkermansia* spp., *Lactobacillus* spp., and *Bifidobacterium* spp., microbes linked to improved metabolic and cognitive outcomes [217,274]. For example, a recent review highlighted that cocoa–microbiota interactions can enhance cognitive function because these bacteria produce defined metabolites (e.g., certain phenolic acids and SCFAs) that modulate host signaling relevant to the brain functions. Indeed, the diversity and composition of one’s gut microbiota determine the yield of bioactive flavanol metabolites, which likely explains inter-individual differences in cognitive responses to flavanol intake [275].

Cognitive improvements observed with flavanol interventions have been associated with microbiota-driven mechanisms. In randomized trials, high-flavanol cocoa supplementation improved memory, executive function, and processing speed in older adults. Notably, chronic cocoa intake in young adults not only enhanced cognitive performance but also led to increased levels of neurotrophins like brain-derived neurotrophic factor (BDNF), a key biomarker of neuroplasticity and learning [217]. This neurotrophic upregulation may be partly downstream of microbiota metabolites [276]. Correspondingly, polyphenol-driven increases in cerebral blood flow have been documented (e.g., after acute cocoa or green tea intake), suggesting improved neurovascular coupling, potentially aided by nitric oxide modulation via gut-derived phenolics. Overall, flavanols illustrate how a polyphenol can bridge the gut and brain, acting as substrates for microbial fermentation, enriching beneficial microbes, reducing microbial toxins, and yielding metabolites and systemic changes that correlate with sharper cognitive function [277].

### 6.4. Anthocyanins and Microbiota-Mediated Cognitive Benefits

Anthocyanins, the pigmented flavonoids in berries (blueberries, strawberries, blackcurrants, etc.), have a similar dual effect on gut microbiota and brain health. These compounds are partially absorbed in the small intestine, up to ~35%, and the remaining majority passes to the colon [278]. This process has several consequences beneficial to the host; anthocyanin intake modulates the composition of gut microbiota, generally increasing the abundance of beneficial microbes (probiotics and SCFA producers) and decreasing potentially pathogenic bacteria. For instance, anthocyanin supplementation has been shown to stimulate growth of *Bifidobacterium* spp. and *Lactobacillus* ssp., while inhibiting opportunistic pathogens like *Staphylococcus aureus* and *Salmonella* ssp [279]. This rebalancing of the microbiota can improve gut ecosystem diversity and reduce inflammatory burden: anthocyanins even reduce endotoxin (lipopolysaccharide) release from Gram-negative bacteria. Additionally, anthocyanin-fermenting microbes (including *Roseburia* spp., *Faecalibacterium* spp., *Parabacteroides* spp.) boost SCFAs production, mainly acetate, propionate, and butyrate, which strengthens the gut barrier and exerts anti-inflammatory and neuromodulator effects. Human trials have reported that dietary berries improve memory and executive function in older adults. For example, 12 weeks of daily blueberry supplementation in an elderly population led to significantly better verbal learning and task-switching performance compared to a placebo [258]. These cognitive improvements coincide with biological signals of neuroprotection. For instance, anthocyanin-rich interventions are associated with reduced oxidative stress and inflammation in the brain, increased neurotrophic factors, and even altered brain activation patterns on fMRI (functional Magnetic Resonance Imaging) [280]. Microbiota-derived metabolites of anthocyanins likely contribute to these effects. Phenolic acids (protocatechuic acid, vanillic acid, etc.) produced by gut bacteria from anthocyanins can cross into circulation and have been shown to exert neuroprotective properties (e.g., by activating antioxidant response pathways and reducing microglial activation) [281]. Moreover, the prebiotic action of anthocyanins, also referred to as “anthocyanins carrying phenols to the colon” for microbial utilization, helps maintain a gut environment that is conducive to brain health and is characterized by lower peripheral inflammation and improved synthesis of vitamins and cofactors by gut microbes [272,281].

### 6.5. Stilbenes and Gut Microbial Metabolites in Cognition

Stilbenoid polyphenols, exemplified by resveratrol, also demonstrate microbiota-mediated effects on cognition and brain aging. Resveratrol gets readily absorbed in the small intestine, but it undergoes extensive metabolism, both by gut microbiota and by host phase II enzymes, yielding metabolites such as dihydroresveratrol and lunularin. Notably, these resveratrol-derived metabolites have antimicrobial activity against pathogenic bacteria, including *Salmonella enterica*, *Escherichia coli*, and *Enterococcus faecalis* [276,282]. By suppressing some deleterious microbes, resveratrol may help reshape the gut microbiota in favor of a healthier balance. This antimicrobial and modulatory action can reduce gut-derived inflammation (for example, lowering endotoxin-producing bacteria) and improve gut barrier integrity, thereby indirectly benefiting brain function. Indeed, alterations in gut microbiota are thought to be one pathway by which resveratrol exerts neuroprotective effects [283].

**Figure 2:**
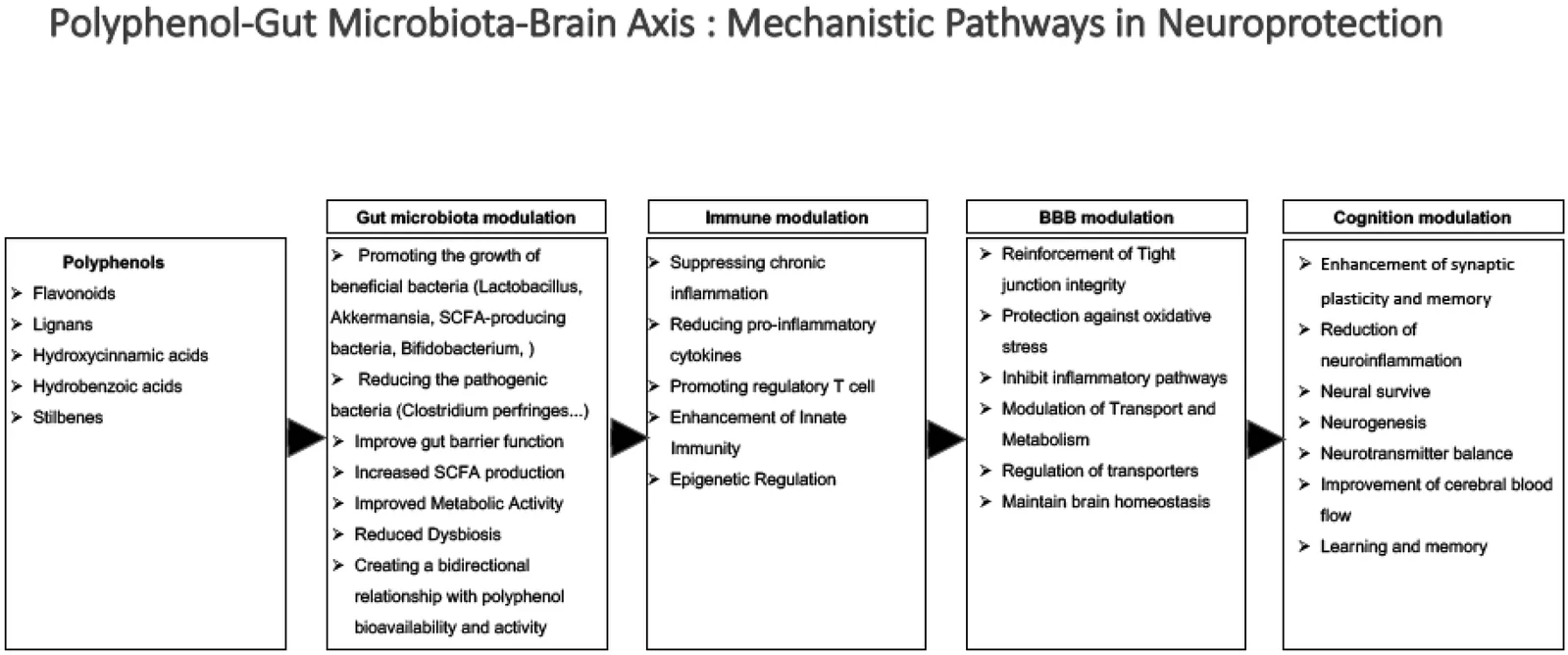
Schematic representation of the mechanistic framework between polyphenols and cognitive function.

The neuroprotective effects and mechanism of Resveratrol on cognitive biomarkers were consolidated by outcomes demonstrated in both animal models and humans. In human trials, resveratrol has shown biological “target engagement” in the CNS. A one-year study in patients with mild Alzheimer’s found that high-dose resveratrol stabilized key Alzheimer’s biomarkers (with less decline in cerebrospinal fluid and Aβ_42_ levels) [284] (Figure 2). Other trials, in healthy adults, observed increased cerebral blood flow during cognitive testing after acute resveratrol intake. Although the direct cognitive improvements in humans have shown contradictory results (some acute studies show improved neurovascular responses without immediate performance gains) [260], the convergence of evidence points to resveratrol’s role in creating a brain-favorable environment for cognitive functions. Part of this may stem from its gut microbiota interactions. By modulating gut flora and producing anti-inflammatory microbial metabolites, resveratrol helps lower the systemic inflammation and oxidative stress that impair cognition [276]. In essence, resveratrol acts as a caloric-restriction mimetic and neuroprotective agent, and the gut microbiota’s processing of resveratrol is integral to its full spectrum of benefits [284].

### 6.6. Curcuminoids (Curcumin) and Microbiota-Driven Neuroprotection

Curcumin, essentially obtained from turmeric, is well known for its anti-inflammatory and neuroprotective effects, but it has very low oral bioavailability. Interestingly, curcumin’s interaction with the gut microbiota appears to overcome this limitation and mediate its cognitive benefits. In the colon, gut microbes metabolize curcumin into more bioactive derivatives (e.g., dihydrocurcumin, tetrahydrocurcumin, ferulic acid, etc.), which are more easily absorbed than the parent compound [285]. Conversely, curcumin itself influences the composition of the gut microbiota. Studies show that oral curcumin can reshape the gut microbial community, promoting the growth of beneficial bacteria while suppressing potentially harmful ones. For example, curcumin intake has been associated with increased abundance of probiotic genera like *Bifidobacterium* spp. and *Lactobacillus* spp., and a reduction in opportunistic or pro-inflammatory groups such as *Enterobacteria* spp. and *Prevotellaceae* spp., thus improving the overall gut microbial balance [286]. Moreover, several curcumin metabolites generated by gut microbes possess neuroactive properties. For instance, tetrahydrocurcumin is a more potent antioxidant than curcumin and exhibits neuroprotective and anti-inflammatory effects in models of neurodegeneration. Therefore, curcumin’s therapeutic potential for the brain seems to rely on this two-way partnership with gut microbes: curcumin shapes a more neuroprotective microbiome and the microbiome in turn transforms curcumin into molecules that can act along the gut–brain axis [287]. Curcumin also exerts beneficial effects on stress-related disorders and depression by modulating inflammatory and endocrine pathways. Clinical evidence demonstrates that curcumin supplementation reduces cortisol levels and pro-inflammatory mediators such as MCP-1 and TNF-α, supporting its role in the regulation of stress and anxiety-related neuropsychiatric conditions [288].

Human studies, while indirect, also support curcumin’s gut-mediated cognitive benefits. In a randomized 18-month trial, middle-aged and older adults taking a daily bioavailable curcumin supplement showed significantly better memory and attention performance than a placebo, along with brain positron emission tomography imaging that indicated lower accumulation of amyloid plaques and tau tangles in memory-related regions [262]. Another trial reported that even a short-term curcumin regimen can enhance cognition in healthy older adults. Just four weeks of highly bioavailable curcumin (Longvida™) led to improved working memory and mood (e.g., reduced fatigue, tension, and stress) compared to a placebo [263]. Although these clinical trials did not measure gut microbiota, the consistent anti-inflammatory and antioxidant effects observed with curcumin suggest involvement of the gut–brain axis. Indeed, researchers have noted that curcumin’s systemic anti-inflammatory action in rodents engages the cholinergic anti-inflammatory pathway and requires intact gut–brain communication. In a rat arthritis model, oral curcumin reduced joint inflammation by boosting VN activity (which triggers an anti-inflammatory reflex), but this benefit was abolished if the VN was surgically cut (vagotomy) or if nicotinic acetylcholine receptors were blocked [289]. In other words, curcumin’s inflammation-lowering effect depended on a functional VN signaling from gut to brain, underscoring that curcumin works through gut-mediated pathways. Taken together, curcumin exemplifies a polyphenol that delivers neuroprotection indirectly, by reshaping the gut microbiota into a butyrate-rich, anti-inflammatory profile. It attenuates peripheral and central inflammation and bolsters the molecular hallmarks of cognitive resilience (less protein aggregation in the brain, improved synaptic function, and enhanced neurotransmitter levels). This highlights the potential of targeting the microbiome to amplify curcumin’s therapeutic effects on the brain [262,289].

### 6.7. Polyphenol-Rich Dietary Patterns and Microbiota-Linked Cognitive Modulation

Beyond individual polyphenols, dietary patterns rich in diverse plant-based compounds like the MeDi have been consistently linked to improved gut microbiota composition and cognitive resilience during aging. Characterized by high intake of fruits, vegetables, whole grains, legumes, olive oil, moderate fish, and wine, the MeDi provides a broad spectrum of polyphenols (e.g., flavonoids, phenolic acids, stilbenes) alongside prebiotic fiber. These components selectively promote beneficial gut microbes such as *Bifidobacterium* spp., *Faecalibacterium* spp., and *Roseburia* spp., which ferment fiber and polyphenols to produce SCFAs, including butyrate metabolites known to support gut integrity, reduce inflammation, and influence neuroimmune and endocrine pathways relevant to brain function [290,291].

Clinical studies have shown that adherence to the MeDi is associated with greater microbial diversity, reduced abundance of pro-inflammatory taxa, and lower levels of circulating inflammatory markers such as C-reactive protein (CRP) and interleukin-6 (IL-6) [292]. Trials like PREDIMED and NU-AGE have demonstrated that these microbial and systemic changes correlate with better metabolic profiles, such as improved lipid levels and insulin sensitivity, and slower cognitive decline [265,293]. Specifically, older adults following a MeDi supplemented with extra-virgin olive oil or nuts showed better memory and executive function over time compared to low-fat diet controls [264].

Experimental models further underscore the microbiota–brain link. A recent rodent study compared a Mediterranean-style versus Western diet and found that rats on the MeDi performed significantly better in learning and memory tasks, with cognitive differences closely mirroring gut microbial shifts. Notably, specific bacterial taxa such as *Candidatus Saccharimonas* were positively associated with improved memory performance. These findings support the emerging paradigm that diet-induced microbiota remodeling shapes systemic and neurochemical environments to protect the brain, operating along a “food–microbiota–brain” axis [151].

### 6.8. Limitations in Polyphenol–Cognition Research

While growing research evidence points to the cognitive benefits of polyphenols through their interactions with the gut–microbiota–brain axis, a number of critical limitations temper these conclusions. A substantial share of the existing evidence comes from animal studies, which fail to fully mirror human physiology. Species-level differences in gut microbiota, metabolism, immune function, and environmental exposure restrict how confidently these results can be applied to humans. This concern is further amplified by the widespread use of germ-free or antibiotic-depleted animal model conditions that are physiologically extreme and largely artificial. Beyond this, a marked tendency toward the publication of statistically significant results raises serious concerns about reporting bias. Funnel plot asymmetry in meta-analyses of polyphenol-related cognitive outcomes suggests that null or negative animal findings are systematically underreported, generating an exaggerated sense of consistency and effect size that is not reliably reproduced in clinical trials. Compounding this, the majority of animal models employed, whether chemically induced or transgenic, such as 5xFAD, APP/PS1, or MPTP variants, recapitulate only isolated features of neurodegeneration and bear limited resemblance to the multifactorial nature of human dementia. Cognitive improvements recorded in these models must therefore be interpreted with considerable restraint.

Bioavailability presents another persistent obstacle. The majority of dietary polyphenols are poorly absorbed in the small intestine and undergo extensive microbial transformation, yielding metabolite profiles that differ markedly between individuals [225,294]. As a result, the concentrations used to demonstrate effects in cell-based experiments often far exceed what is realistically achievable in vivo. Dosing inconsistencies across studies add a further layer of complexity: experimental protocols routinely administer isolated polyphenols at quantities far above normal dietary levels, whereas real-world human consumption occurs at lower doses and within the context of complex food matrices [295]. Rodent studies, for instance, commonly use doses of 50 to 500 mg/kg/day amounts that, when converted to human equivalents, far exceed what diet or supplementation can safely provide. No consistent dose–response curve has been established for any polyphenol class in relation to cognitive endpoints, and the absence of validated biomarkers capable of confirming central nervous system exposure means that it remains difficult to determine whether observed cognitive effects are genuinely attributable to polyphenol activity or to non-specific consequences of dietary change.

In the clinical domain, the strength of available evidence varies widely, and a clear distinction must be maintained between study designs capable of establishing causation and those limited to identifying associations. Randomized controlled trials, while methodologically superior for causal inference, are themselves beset by recurring weaknesses: most enroll fewer than one hundred participants, run for no longer than eight to twenty-four weeks, a timeframe insufficient to capture change in slowly progressing neurodegenerative conditions, and frequently lack adequate blinding. Many rely on mixed polyphenol extracts from which no single bioactive component can be individually attributed. A particularly consequential oversight is the failure to stratify participants by gut microbiota metabotype prior to enrollment, meaning that high metabolite producers of equol or urolithins are grouped alongside non-producers, effectively diluting effect sizes and generating apparent heterogeneity [214].

A final and fundamental gap concerns the absence of a coherent theoretical framework that positions the gut microbiota as a primary independent variable rather than a secondary contextual factor. The prevailing clinical trial design treats polyphenol supplementation as the intervention and microbiota modulation as the outcome, without accounting for baseline microbial composition or selecting for likely responders. Future research should be structured around the polyphenol–microbiota axis as its organizing principle, stratifying participants by metabotype at baseline, designating microbiota-derived metabolites as primary rather than exploratory outcomes, and deploying integrative multi-omic approaches (metagenomics, metabolomics, neuroimaging) to trace the full mechanistic chain from dietary polyphenol intake through microbial biotransformation to neuroprotective effect in the brain. Without trials built around such a rigorous, hypothesis-driven framework, the field will remain anchored in descriptive science, unable to generate the actionable clinical recommendations that the evidence base has long promised but not yet delivered.

## 7. Conclusions

A growing body of evidence highlights the important role of dietary polyphenols in regulating the gut–microbiota–brain axis and shaping cognitive function. Preclinical research consistently shows that polyphenols and their microbiota-derived metabolites, such as phenolic acids, urolithins, and short-chain fatty acids, exert neuroprotective effects. These actions are mediated through antioxidant activity, attenuation of neuroinflammatory pathways, and enhancement of synaptic plasticity, particularly via BDNF/CREB signaling. Importantly, the gut microbiota acts as a key bioactivation system, converting poorly absorbed dietary compounds into more lipophilic metabolites capable of crossing the blood–brain barrier and exerting effects within the central nervous system.

However, despite strong mechanistic evidence, findings from human clinical studies remain limited, heterogeneous, and often inconclusive. These inconsistencies are largely attributable to variations in study design, sources and doses of polyphenols, and critically, interindividual differences in gut microbiota composition. Individuals possessing microbial communities capable of producing bioactive metabolites such as equol or urolithin A tend to exhibit greater cognitive benefits, indicating that microbiota composition is a key determinant of response rather than merely a confounding factor. Additional challenges include the low bioavailability of polyphenols, the lack of standardized biomarkers, and the limited integration of microbiome and metabolomics data, all of which hinder comparability across studies.

Future investigations should focus on well-designed, adequately powered randomized controlled trials that simultaneously assess cognitive outcomes, gut microbiota composition, circulating metabolites, and neuroimaging markers. Establishing standardized dosing regimens, ensuring precise chemical characterization of polyphenol interventions, and advancing personalized nutrition approaches such as targeted prebiotic and probiotic strategies will be critical for successful clinical translation. Ultimately, the major challenge in this field is no longer understanding the mechanisms but translating consistent preclinical findings into robust clinical evidence that can inform dietary guidelines and support polyphenol-based strategies for the prevention of cognitive decline and dementia.

## Data Availability

The data are available upon request to the corresponding author.
