# Correction: Sbai et al. The Role of Polyphenols on Cognitive Function and Dementia Through Gut–Microbiota–Brain Axis Modulation: A Narrative Review. *Nutrients* 2026, *18*, 1697

**DOI:** 10.3390/nu18213598

**Published:** 2026-10-30

**Authors:** Oualid Sbai, Aishwarya Chaure, Lorena Perrone, Patrick Poucheret

**Affiliations:** 1 Laboratory of Transmission, Control and Immunobiology of Infections (LTCII), LR11IPT02, Institut Pasteur de Tunis, Tunis 1068, Tunisia; oualid.sbai@pasteur.tn; 2 Department of Food and BioResource Technology, Faculty of Tropical AgriSciences, Czech University of Life Sciences Prague, 165 00 Prague, Czech Republic; chaure@ftz.czu.cz; 3 Department of Life Sciences, Health and Health Professions, Link Campus University, 00165 Rome, Italy; 4 Institute of Endotypes in Oncology, Metabolism and Immunology “G. Salvatore” (IEOMI), Consiglio Nazionale delle Ricerche (CNR), 80100 Naples, Italy; 5 Univ Montpellier, CIRAD, Montpellier SupAgro, Univ Avignon, Univ La Réunion, IRD, 34090 Montpellier, France; patrick.poucheret@umontpellier.fr

**Addition of an Author**

Aishwarya Chaure was not included as an author in the original publication [1].

Aishwarya Chaure was inadvertently omitted from the author list of the original publication due to an oversight during the manuscript submission process. She contributed to the article search and writing of the manuscript and therefore should have been included as an author. The Author Contributions statement has been corrected accordingly. 

“**Author Contributions:** O.S., A.C., L.P. and P.P. performed the article search and wrote the article; O.S. prepared Figures 1 and 2; A.C. and P.P. prepared Table 1; L.P. and P.P. revised the manuscript. All authors have read and agreed to the published version of the manuscript.”

The newly added address for Aishwarya Chaure is:

^2^ Department of Food and BioResource Technology, Faculty of Tropical AgriSciences, Czech University of Life Sciences Prague, 165 00 Prague, Czech Republic; chaure@ftz.czu.cz

The authors confirm that the scientific conclusions of the article remain unaffected. This correction was approved by the Academic Editor. The original publication has al-so been updated.

## References

[1] Sbai O., Chaure A., Perrone L., Poucheret P. (2026). The Role of Polyphenols on Cognitive Function and Dementia Through Gut–Microbiota–Brain Axis Modulation: A Narrative Review. Nutrients.

